# Redefining Liver Transplantation Indications for Hepatic Malignancies in the Era of Precision Transplant Oncology: An Up-to-Date Narrative Review

**DOI:** 10.3390/jcm15103579

**Published:** 2026-05-07

**Authors:** Mario Romeo, Fiammetta Di Nardo, Carmine Napolitano, Paolo Vaia, Claudio Basile, Giusy Senese, Annachiara Coppola, Patrizia Iodice, Simone Olivieri, Alessandro Federico, Marcello Dallio

**Affiliations:** 1 Hepatogastroenterology Division, Department of Precision Medicine, University of Campania Luigi Vanvitelli, Piazza Miraglia 2, 80138 Naples, Italy; mario.romeo@unicampania.it (M.R.); fiammetta.dinardo@unicampania.it (F.D.N.); carmine.napolitano@unicampania.it (C.N.); paolo.vaia@unicampania.it (P.V.); claudio.basile@unicampania.it (C.B.); giusy.senese@unicampania.it (G.S.); annachiara.coppola@unicampania.it (A.C.); simone.oliveri@unicampania.it (S.O.); alessandro.federico@unicampania.it (A.F.); 2 Division of Medical Oncology, AORN Azienda dei Colli, Monaldi Hospital, via Leonardo Bianchi, 80131 Naples, Italy; patrizia.iodice@ospedalideicolli.it

**Keywords:** transplant oncology, liver cancer, precision medicine

## Abstract

**Background:** Hepatic malignancies are a major global health burden, with rising incidence, high mortality, and frequent diagnosis at advanced or unresectable stages. Although surgical resection, locoregional therapies, and systemic treatments have improved outcomes, many patients remain ineligible for curative strategies because of tumor burden, anatomical constraints, or liver dysfunction. Liver transplantation (LT) has therefore evolved from a treatment limited to selected hepatocellular carcinoma (HCC) cases within strict morphological criteria to a broader oncologic option guided by tumor biology and treatment response. This review provides an updated overview of the expanding role of LT in hepatic malignancies and the transition toward precision transplant oncology. **Methods:** We conducted a narrative review of current evidence on LT in HCC, cholangiocarcinoma (CCA), and colorectal liver metastases (CRLM), focusing on candidate selection, neoadjuvant strategies, molecular profiling, immunological aspects, and future perspectives. **Results:** In HCC, expanded criteria and bridging/downstaging strategies, including immunotherapy, have increased transplant eligibility, although concerns remain regarding rejection risk and post-transplant management. In CCA, especially perihilar disease, standardized neoadjuvant protocols followed by LT have achieved encouraging long-term survival in highly selected patients, whereas intrahepatic CCA remains investigational within prospective biomarker-driven studies. In CRLM, once considered an absolute contraindication, recent evidence supports LT in selected patients with liver-confined and biologically favorable disease, emphasizing the importance of tumor kinetics, molecular features, and response to systemic therapy. **Conclusions:** Integration of molecular oncology, immunology, and advanced therapies is redefining LT indications for hepatic malignancies. Future progress will depend on biomarker-driven selection, precision medicine, and multidisciplinary decision-making to optimize outcomes while addressing ethical challenges in organ allocation.

## 1. Background

### 1.1. Global Burden of Liver Malignancies: Is It Time to Expand the Frontiers of Liver Transplantation?

Liver cancer represents a substantial global public health challenge, with high incidence and mortality across diverse geographical regions. According to the most recent estimates, primary liver cancers (PLCs), principally embracing hepatocellular carcinoma (HCC) and cholangiocarcinoma (CCA), are one of the most common malignancies worldwide, ranking among the top six cancers by incidence and the third leading cause of cancer-related death in 46 countries, with an estimated 905,700 new cases and 830,200 deaths in that year [1]. Dramatically, projections suggest a significant escalation in the global burden of PLC over the next two decades, when the number of new cases is expected to rise by 55.0%, reaching an estimated 1.4 million annual diagnoses. This trend is mirrored by a projected 56.4% increase in mortality, potentially resulting in 1.3 million deaths [1].

Among the PLCs, HCC represents approximately 75–85% of cases worldwide [2]. This dominance reflects the strong association between HCC and chronic liver disease, particularly chronic hepatitis B and C infection, which remains a major etiological driver in many high-incidence regions. In other countries, however, beyond the established role of viral etiologies, the escalating incidence of HCC is increasingly attributable to the surge in Metabolic Dysfunction-Associated Steatotic Liver Disease (MASLD) cases [3]. This etiological transition is particularly noteworthy as MASLD-associated hepatocarcinogenesis frequently bypasses the traditional cirrhosis-to-cancer sequence, complicating current surveillance protocols and patient management [4].

Cholangiocarcinoma (CCA), a malignancy arising from the biliary epithelium, represents the second most common primary liver tumour, albeit at a much lower proportion than HCC [5]. It accounts for roughly 10–15% of primary liver cancers globally, and its incidence appears to be increasing in certain populations. Notably, CCA is often diagnosed at an advanced stage and has a generally poor prognosis, underscoring the clinical challenges it presents [6].

Beyond HCC and CCA, the liver may rarely harbor unusual primary malignancies, including primary squamous cell carcinoma of the liver (PSCCL), an exceptionally rare entity associated with aggressive clinical behavior and poor prognosis [7]. Its pathogenesis remains incompletely understood, although chronic inflammation, congenital hepatic cysts, hepatolithiasis, and squamous metaplasia of biliary epithelium have been proposed as potential predisposing factors [7]. Diagnosis is challenging and requires exclusion of metastatic squamous cell carcinoma from extrahepatic primary sites. Owing to the rarity of this tumor, no standardized management exists; however, surgical resection, systemic chemotherapy, and multimodal approaches have been reported in selected cases [7]. Current evidence remains limited to case reports and small series, highlighting the need for greater biological characterization and individualized multidisciplinary management [7,8,9].

While PLCs are themselves a major cause of morbidity and mortality, secondary liver malignancies constitute an even greater oncological burden [10]. The rich dual blood supply in the liver explains why this is a common site for haematogenous dissemination of extracolonic malignancies, and metastatic disease to the liver is far more common than primary hepatic malignancy [11]. Among these, colorectal cancer (CRC) represents the single most frequent source of liver metastases. CRC itself is a leading global cancer, and between 20% and 30% of patients develop clinically significant colorectal liver metastases (CRLM) at some point during their disease course, constituting the principal cause of death in many of these individuals [12]. The high prevalence of CRC liver metastases reflects not only the biological proclivity of colorectal tumours to spread via portal circulation to the liver, but also the global burden of CRC itself, which remains one of the most diagnosed cancers worldwide.

Beyond their epidemiological relevance, these trends carry direct therapeutic implications. The rising incidence of primary and secondary liver malignancies persists despite major advances in surveillance, surgical techniques, locoregional treatments, and systemic therapies. As a result, a substantial proportion of patients still present with unresectable disease, advanced tumour burden, or relapse after conventional treatment. Consequently, the growing global burden of hepatic malignancies should not be interpreted solely as a public health statistic, but also as evidence of an expanding population with unmet curative needs. In this context, liver transplantation (LT) warrants reconsideration not only as rescue therapy for selected HCC cases, but as a potentially broader oncologic strategy for carefully chosen patients in whom tumour biology, disease confinement, and treatment response suggest a realistic chance of long-term survival.

### 1.2. Aim of the Review

Collectively, the epidemiology of primary and secondary liver malignancies emphasizes the enormous clinical and public health impact of hepatic tumours and the urgent need for improved diagnostic, preventive, and therapeutic strategies. In this context, in recent decades, the role of LT, first limited to end-stage liver disease and selected primary liver cancers, has been explored and is progressively evolving, expanding this approach as a precious additional alternative for carefully selected patients in settings where conventional medical therapy and surgical resection fail to achieve durable control and prolonged survival.

Despite major advances in hepatic surgery, locoregional treatments, and systemic therapies, the management of liver malignancies remains constrained by important biological and technical limitations. Together, the recognition of the limitations of conventional treatments, simultaneously with the improved understanding of tumour biology, has provided the rationale for reconsidering and progressively expanding the role of LT in selected hepatic malignancies.

This review, after presenting the evolving paradigm of transplant oncology and the relative supporting rationale, examines the expanding role of LT in hepatobiliary malignancies, overviewing tumour biology, emerging targets, current clinical protocols for HCC, CCA, and CRLMs, as well as future directions in this field.

## 2. Rationale for Redefining the Role of Transplant in the Management of Hepatic Malignancies

### 2.1. Limitations of Surgical Resection and Locoregional Therapies in Managing Hepatic Malignancies

Surgical resection has long been established as the gold standard curative approach for PLCs, particularly for HCC and for selected CRLMs, offering the highest long-term survival rates when complete tumour clearance with negative margins is achievable [13]. However, despite advances in surgical techniques and perioperative care, significant limitations restrict its applicability and effectiveness in a large proportion of patients [14].

Resectability is limited by factors that could be patient-related and tumour-related. Many patients are deemed ineligible due to underlying liver dysfunction, particularly cirrhosis in HCC, poor general performance status, or significant comorbidities that increase perioperative risk. In patients with cirrhosis, only a limited proportion can safely tolerate major hepatic resection without precipitating postoperative liver failure, and current series suggest that even in experienced centres, a minority of HCC patients constitute true surgical candidates [15]. Additionally, tumour features such as lesion size, multifocality, bilobar involvement, or proximity to major vascular structures can preclude complete resection without compromising adequate future liver remnant (FLR) [15].

A particularly challenging scenario is represented by HCC with portal vein tumour thrombosis (PVTT), historically associated with poor prognosis and often considered unsuitable for curative surgery. However, growing evidence suggests that surgical resection may provide meaningful survival benefit in carefully selected patients, particularly those with preserved liver function, limited intrahepatic tumor burden, and segmental or lobar rather than main trunk portal vein involvement [16]. In such cases, hepatectomy with thrombectomy or en bloc resection may be considered within experienced centers and multidisciplinary programs [17]. Nevertheless, outcomes remain strongly dependent on the extent of vascular invasion, underlying liver reserve, and the availability of adjunctive locoregional or systemic therapies, underscoring the need for individualized strategies [17].

Beyond technical resectability, postoperative liver regeneration remains a crucial determinant of outcomes after partial hepatectomy.

In this context, innate immune signaling, particularly through Toll-like receptors (TLRs), plays an important regulatory role in the early regenerative response [18,19].

Following hepatic resection, damage-associated molecular patterns and gut-derived microbial products activate Kupffer cells and other non-parenchymal cells through TLR-mediated pathways, promoting the release of cytokines such as Tumor Necrosis Factor-α (TNF-α) and interleukin-6 (IL-6), which prime hepatocytes to re-enter the cell cycle [18,19]. Experimental studies suggest that appropriate TLR signaling is essential for effective regeneration, whereas excessive or dysregulated activation may contribute to inflammation, impaired recovery, and postoperative liver dysfunction [18,19]. These observations further highlight the importance of host biology, beyond anatomical criteria alone, in determining the feasibility and success of surgical strategies.

For CRLM, although surgical resection can offer a potential cure, only around 20% of patients are initially eligible based on oncological and technical criteria, with the remainder requiring systemic therapy or locoregional strategies in an attempt at downsizing [20]. Even among resectable cases, extensive procedures such as two-stage hepatectomy with portal vein embolization or combined resections carry an increased operative risk and are not universally feasible [21].

The clinical management of patients unsuitable for surgical resection has been significantly bolstered by the development of locoregional therapies, including radiofrequency and microwave ablation (RFA/MWA), transarterial chemoembolization (TACE), transarterial radioembolization (TARE), and stereotactic body radiotherapy (SBRT). These modalities offer a less invasive means of achieving local tumor control, often serving as a crucial bridge or downstaging strategy before transplantation, although their curative potential is inherently limited by several technical and biological factors [22]. In this sense, the efficacy of thermal ablation is highly dependent on tumor size and location, with the success rates diminishing sharply for lesions exceeding 3 cm or those situated near major vessels, where the “heat sink effect” often leads to incomplete necrosis [23].

Furthermore, anatomical challenges such as proximity to the hepatic dome or gallbladder frequently result in higher recurrence rates compared to the radicality of surgical resection [24]. This discrepancy is reflected in meta-analyses showing that locoregional approaches generally yield inferior disease-free survival and higher local progression in resectable settings [25]. These constraints are even more pronounced in multifocal disease or cases with extrahepatic involvement, where TACE may provide palliation without significant benefits on the prognosis.

Although generally well tolerated, advanced locoregional approaches may also be associated with rare but potentially severe complications. In particular, ablative radiation lobectomy has been linked to isolated reports of hepatocolic fistula formation, likely resulting from radiation-induced necrosis, inflammatory adhesions, and subsequent transmural injury involving adjacent colonic segments [26].

Although uncommon, this complication may present with abdominal pain, fever, sepsis, or enteric contamination of the liver cavity and usually requires prompt multidisciplinary management, including imaging evaluation, antibiotic therapy, drainage, and, in selected cases, surgical intervention [26]. Awareness of this rare event is relevant when treating lesions close to the hepatic flexure or in patients with prior abdominal surgery, where altered anatomical relationships may increase the risk of collateral injury.

Within this evolving therapeutic landscape, advanced imaging techniques are increasingly contributing to treatment personalization. In particular, 18F-fluorodeoxyglucose positron emission tomography/computed tomography (18F-FDG PET-CT)-based radiotherapy planning may enhance target volume delineation by integrating metabolic and anatomical information, thereby reducing geographic miss and enabling more precise radiation delivery [27].

PET-CT may also identify occult intrahepatic or extrahepatic disease not evident on conventional imaging, refine patient selection, and provide prognostic information through metabolic response assessment [27]. These advantages are particularly relevant in biologically heterogeneous tumors, where functional imaging may complement morphology-based evaluation and support more individualized oncologic locoregional strategies.

Altogether, the above-presented limitations of current locoregional techniques underscore the necessity of a multidisciplinary approach and highlight a critical unmet need for integrating systemic agents and targeted therapies to improve outcomes for patients with borderline-resectable disease.

### 2.2. Exploring the Unmet Clinical Need in Unresectable Biologically Favorable Disease

As previously discussed, many factors, including features exclusively related to the patient and his comorbidities, may impact tumour unresectability even in a biologically favourable malignancy. Consequently, in clinical practice, a substantial portion of patients without an aggressive disease biology remain ineligible for curative resection due to anatomical, functional, or technical constraints, and these types of patients form a large subgroup that represents a major unmet clinical need in hepatobiliary oncology.

In the context of PLC, an indolent biology could be characterised by limited tumour burden, absence of macrovascular invasion, favourable tumour markers (e.g., low alpha-fetoprotein (AFP) in HCC), and preserved performance status, suggesting potential for durable disease control if curative options could be safely extended. However, a significant portion of patients remains in the “grey zone” with the preclusion of a resection, based on the current surgical criteria, and locoregional therapies that may be ineffective or infeasible for certain tumours due to size or anatomical proximity to major vasculature or biliary structures, despite underlying biology that might otherwise suggest good long-term outcomes [28].

In CRLM, a disease characterised by a limited metastatic burden and a favourable response to systemic chemotherapy, which often achieves excellent disease control, could remain technically unresectable for different factors, such as a bilobar distribution or an unfavourable segmental involvement. These patients frequently demonstrate prolonged progression-free intervals and may have biological indicators of slower disease progression, yet current standards often consign them to palliative strategies, despite relatively favourable prognostic features [29].

To overcome this unmet need, the recent interest in this field is driven by alternative approaches to offer potentially curative treatment to patients whose disease is biologically amenable but surgically inaccessible, such as downstaging and bridging strategies utilising modern systemic therapies (immunotherapy, targeted agents) in combination with locoregional treatments to convert unresectable disease into resectable or transplantable status and using multimodal protocols integrating systemic and locoregional approaches to consolidate disease control while preserving organ function [30].

Another key point that is driving the shift in the traditional paradigm in this field is the extension of the criteria for LT beyond the traditional HCC guidelines, such as the Milan criteria, to include selected patients with HCC exhibiting favourable biological behaviour, or even selected cases of CRLM with excellent systemic disease control.

### 2.3. Historical Contraindication to Liver Transplant and Paradigm Shifts in PLC

Currently, LT is considered the gold standard treatment in early-stage HCC patients unsuitable for resection, because it acts on both tumour removal and underlying liver cirrhosis. However, historically, malignant disease was considered a contraindication to LT because of high recurrence rates, poor long-term survival, and organ scarcity. A major paradigm shift occurred with the introduction of the Milan criteria, which demonstrated that carefully selected patients with early HCC could achieve post-transplant outcomes comparable to non-oncologic indications [31,32].

In this field, the landmark shift began with the realisation that selected tumours with a defined biological behaviour could achieve acceptable post-transplant outcomes if appropriately stratified, and the first milestone in this field was the introduction of the Milan criteria for small and unresectable HCCs in cirrhotic patients. The morphological limits for these criteria are a single tumour ≤ 5 cm or up to three tumours each ≤ 3 cm, with no macrovascular invasion or extrahepatic disease, and with a low recurrence rate and 5-year overall survival rates comparable to those of LT in non-oncological patients [31]. The rigorous caution of the Milan era was largely a reaction to the lessons learned from early failures in transplant oncology. In the pre-Mazzaferro period, LT was often attempted for advanced or symptomatic malignancies, leading to early recurrence, which frequently occurred within the first year, and dismal survival rates that were hard to justify given the scarcity of donor organs.

Importantly, the historical concern regarding organ scarcity has been progressively reshaped by innovations in donor management and graft preservation [33]. The increasing use of extended-criteria donors, donation after circulatory death (DCD), and ex situ machine perfusion technologies has significantly expanded the potential donor pool while improving graft assessment and post-transplant outcomes [34].

In particular, normothermic and hypothermic machine perfusion have enhanced organ viability, reduced ischemia–reperfusion injury, and enabled safer utilization of marginal grafts [34]. These advances may provide an additional ethical and logistical rationale for considering transplantation in carefully selected oncologic indications, as broader graft availability could reduce the traditional tension between utility and equity in organ allocation [33]. In parallel with these developments in organ availability, efforts to refine candidate selection continued to evolve through the validation and progressive expansion of oncologic eligibility criteria. In this way, the original Milan framework was later expanded [35] through models such as the University of California, San Francisco (UCSF) [36], which permitted transplantation for a single tumour up to 6.5 cm, or up to three tumours with a total diameter ≤ 8 cm, without significantly compromising post-transplant survival [37], Up-to-Seven, Metroticket 2.0, and AFP-based criteria, which integrated tumour burden with biological variables while maintaining acceptable survival outcomes [38].

Collectively, these developments established that morphology alone is an imperfect surrogate of tumour aggressiveness and that biologically informed selection is central to modern transplant oncology.

From an ethical perspective, the expansion of LT to oncologic indications raises important considerations regarding organ allocation and distributive justice [39]. Historically, strict selection criteria were justified by the need to maximize utility, prioritizing patients with the highest probability of long-term survival. However, as outcomes in selected oncologic populations have improved, the balance between utility and equity is being progressively re-evaluated [39].

Approaches to this issue vary across transplant systems. In the United States, the MELD-based allocation system incorporates standardized exception points for selected HCC patients, aiming to balance urgency and post-transplant benefit [40,41]. In contrast, several European countries adopt more centre-driven or regionally adapted criteria, often allowing greater flexibility in candidate selection but also introducing variability in access [40,41]. In Asia, where living donor LT is more prevalent, ethical considerations additionally involve donor risk, which may influence the threshold for accepting oncologic indications [42]. These differences highlight that the ethical justification for expanding transplant oncology is not uniform but depends on local organ availability, allocation policies, and societal values [39]. In this context, the ongoing expansion of the donor pool through extended-criteria donors and machine perfusion may help mitigate ethical tensions by increasing organ availability, thereby supporting a more balanced integration of oncologic indications without compromising fairness in allocation [39].

### 2.4. Historical Contraindication to Transplant and Paradigm Shift in Colorectal Liver Metastases

For decades, liver transplantation for CRLMs was considered an absolute contraindication. Early experiences in the 1980s and 1990s were marked by dismal oncologic outcomes, with high rates of rapid recurrence and poor overall survival, reinforcing the perception that metastatic colorectal cancer represented a systemic disease biologically incompatible with transplantation [32].

Initial case series reported recurrence rates exceeding 60–80%, often occurring within the first year following transplantation. At that time, limited understanding of tumour biology, absence of effective systemic chemotherapy, and non-standardised selection criteria contributed to unfavourable outcomes. Furthermore, immunosuppression was thought to accelerate tumour progression by impairing immune surveillance and facilitating micrometastatic outgrowth, intensifying oncologic concerns [43].

These early failures shaped transplant policy for decades. Unlike HCC, where the introduction of strict morphologically redefined eligibility, CRLM lacked a validated biological selection framework [31].

Given organ scarcity and the ethical imperative of utility, allocating grafts to patients with metastatic disease and historically poor survival was considered unjustifiable [44].

However, the oncologic landscape of colorectal cancer has evolved dramatically. Advances in systemic chemotherapy, targeted therapies, and improved molecular stratification have significantly prolonged survival in selected patients with metastatic disease [29]. Simultaneously, a better understanding of tumour burden, response to therapy, and disease kinetics has challenged the simplistic classification of CRLM as uniformly systemic and rapidly lethal, thus leading to a paradigm shift: rather than viewing CRLM as an absolute contraindication, investigators began to question whether carefully selected patients with liver-confined, biologically favourable disease might achieve meaningful survival after transplantation.

The reconsideration of LT for CRLM is rooted in a profound transformation of both oncologic management and biological understanding of metastatic colorectal cancer. While early experiences demonstrated unacceptable recurrence rates, more recent data suggest that a carefully selected subgroup of patients with liver-confined, biologically favourable disease may achieve long-term survival following transplantation [45].

A pivotal turning point emerged from the Norwegian SECA (Secondary Cancer) studies, in which patients with unresectable limited-to-liver CRLM undergoing transplantation achieved a 5-year overall survival of approximately 60%, despite high recurrence rates [46].

Although disease recurrence remained common, many relapses were slow-growing and amenable to secondary curative interventions, fundamentally challenging the historical assumption that post-transplant recurrence inevitably implied rapid mortality. Subsequent refinement of selection criteria in the SECA-II study, seven years later, led to even more favourable outcomes, with 5-year overall survival approaching 83% in highly selected patients [47]. These results highlighted the crucial concept that the prognosis in metastatic colorectal cancer is not uniform but strongly influenced by tumour biology. Factors such as response to systemic chemotherapy, low pre-transplant carcinoembryonic antigen (CEA) levels, limited tumour burden, long disease-free interval from primary tumour resection, and absence of extrahepatic disease have emerged as key predictors of favourable post-transplant outcomes [48]. Importantly, molecular profiling has further refined prognostic stratification, identifying subsets with more indolent disease behaviour [49].

From a surgical oncology perspective, transplantation offers theoretical advantages over conventional resection in patients with extensive bilobar disease. Whereas resection is constrained by remnant liver volume and vascular anatomy, transplantation allows complete removal of the tumour-bearing liver, potentially eliminating occult micrometastatic foci confined to the hepatic parenchyma [50].

In highly selected patients, this radical approach may achieve survival outcomes comparable to those observed in established transplant indications.

Taken together, these advances support a biologically driven rather than dogmatic framework. The question is no longer whether CRLM per se constitutes a contraindication, but which patients exhibit a tumour phenotype compatible with durable survival under post-transplant immunosuppression. This shift from categorical exclusion to precision-based inclusion represents one of the most significant conceptual evolutions in contemporary transplant oncology.

### 2.5. Transplant Oncology: Liver Transplantation at the Interface of Oncology and Immunology

The clinical landscape of LT in oncology has been reshaped by growing recognition that traditional morphologic criteria alone are insufficient to capture the complex interplay between tumour biology, host factors, and long-term outcomes.

The emerging discipline of transplant oncology reflects this shift, underpinned by advances in imaging, molecular profiling, immunology, and therapeutic strategy. The shift from morphologic to biologic selection, the integration of molecular oncology with transplant immunology, and the conceptualisation of precision transplant oncology collectively articulate the rationale for redefining the role of LT in cancer care.

The old morphological criteria were pivotal in establishing transplantation as a curative option for selected patients, but they are inherently limited by their reliance on static, anatomical features. Tumours of similar size may harbour vastly different biological behaviours, and anatomic thresholds alone cannot fully stratify risk of recurrence or systemic spread [51].

The evolution towards biologic selection recognises that factors such as vascular invasion, histopathologic grade, proliferation indices, tumour markers, and dynamic response to therapy provide additional discriminatory power. Incorporating these biologic indicators enhances prognostic precision and allows clinicians to identify patients whose disease is inherently less aggressive and more amenable to curative intervention, even if their morphology falls outside traditional cut-offs.

This shift reflects a broader oncologic trend away from “one-size-fits-all” criteria towards multidimensional assessment of tumour behaviour [52].

The convergence of molecular oncology and transplant immunology represents a transformative frontier in transplant oncology. Molecular profiling techniques, including genomics, transcriptomics, proteomics, and emerging machine learning models, enable the characterization of tumour subtypes, mutational landscapes, and pathways that drive growth, invasion, and therapeutic resistance.

When integrated with transplant decision making, these insights can refine risk stratification beyond conventional clinical and radiologic evaluation. At the same time, advances in understanding the immunological milieu, both the tumour microenvironment and the transplanted organ, have highlighted the dual roles of the immune system in tumour surveillance and transplant tolerance. Immunosuppressive regimens, once chosen primarily to prevent graft rejection, are now being scrutinised for their impact on residual tumour cells and recurrence risk. Synergistic strategies that balance antitumour immunity with graft acceptance, including the selective use of immunomodulatory agents, represent an important interface between oncology and transplant medicine [53]. This integration opens a pathway toward biologically informed immunosuppressive protocols tailored to the oncologic context.

Building on these developments, the emerging paradigm of precision transplant oncology seeks to personalise transplant-based cancer treatment by integrating patient-specific tumour biology, host factors, response to therapy, and immunologic profile into a unified framework [54].

Precision transplant oncology represents an emerging framework that moves beyond eligibility thresholds based solely on tumour size or number. Its goal is to improve the prediction of outcomes and recurrence risk by integrating clinical, radiological, and biological data. In this scenario, artificial intelligence (AI) and advanced image processing are increasingly emerging as key tools in precision liver oncology [55]. In diagnostic imaging, AI-based approaches may support lesion detection, segmentation, characterization, and differentiation between benign lesions, HCC, CCA, and metastatic disease, particularly through radiomics and deep learning applied to CT, MRI, and ultrasound images [55]. Beyond diagnosis, AI may improve treatment planning by refining tumor burden assessment, estimating future liver remnant volume, supporting radiotherapy or locoregional treatment planning, and predicting response to TACE, TARE, ablation, systemic therapy, or immunotherapy [56]. From a prognostic perspective, radiomic and machine-learning models may integrate imaging features with clinical, molecular, and laboratory variables to estimate microvascular invasion, recurrence risk, post-treatment outcomes, and, potentially, post-transplant recurrence [57]. However, despite promising results, clinical implementation remains limited by retrospective study designs, heterogeneity in imaging protocols, lack of external validation, and the need for explainable and reproducible algorithms. Therefore, AI should currently be considered a decision-support tool rather than a substitute for multidisciplinary evaluation [57]. In line with this, a very recent study shows how machine learning models that integrate multiple clinical, radiological, and biological variables improve the prediction of post-LT recurrence compared to traditional criteria [58]. In this evolving context, transplant eligibility may progressively shift from a binary decision based on fixed cutoffs toward a predictive continuum informed by clinical, radiological, and biological variables. Therefore, practically, in this context, a patient with an anatomically borderline tumour but a favourable molecular signature and an excellent response to neoadjuvant therapy might be considered an appropriate candidate according to a precision-based model, while a morphologically small lesion with aggressive molecular characteristics might be downgraded due to the high risk of recurrence.

**Figure 1:**
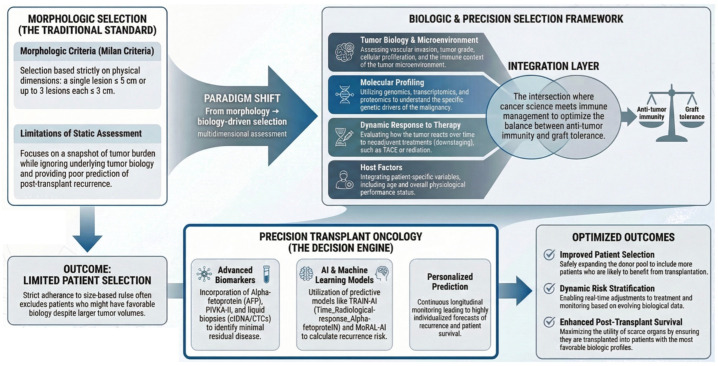
Paradigm shift in Transplant Oncology: from Morphology to Biology-Driven Selection. PIVKA-II: Protein induced by vitamin K absence-II; AI: artificial intelligence; ctDNA: circulating DNA; CTCs: circulating tumor cells.

Collectively, these principles establish a compelling rationale for redefining transplant oncology: by prioritising biologic insight over simple morphology, integrating molecular and immunologic determinants, and embracing precision medicine principles, the field is moving toward more nuanced, personalised, and effective utilisation of LT in cancer treatment (Figure 1).

Nevertheless, it is important to distinguish between concepts already influencing contemporary clinical practice and those that remain investigational.

While response-based candidate selection, multidisciplinary evaluation, and integration of selected biological markers are increasingly shaping real-world transplant decision-making, more advanced frameworks based on multi-omics profiling, AI, and fully individualized predictive models still require robust prospective validation before routine implementation (Figure 1). At present, these approaches should be viewed as promising tools to refine future selection strategies rather than established standards of care.

## 3. HCC: Is It Time to Expand Transplant Eligibility Through Immunological Modulation?

Beyond tumor burden and progression patterns, advanced HCC may occasionally present with paraneoplastic manifestations that reflect aggressive metabolic activity and tumor biology. Among these, non-islet cell tumor hypoglycemia is a rare but clinically relevant complication, more frequently observed in patients with massive tumor burden [59]. Proposed mechanisms include high glucose consumption by rapidly proliferating tumor tissue, impaired hepatic gluconeogenesis due to extensive parenchymal replacement, and, in selected cases, secretion of incompletely processed insulin-like growth factor-2 [60]. Clinically, severe or recurrent hypoglycemia may significantly worsen performance status and require urgent supportive management. Importantly, resolution of hypoglycemia has been reported after effective anticancer treatment, including systemic chemotherapy and tumor resection, supporting its direct relationship with tumor activity and burden [60]. These clinical observations further underscore the marked biological heterogeneity of HCC and provide a rationale for examining the tumor microenvironment and its potential therapeutic targets.

### 3.1. Immunobiology of HCC-Associated Microenvironment: Exploring a Heterogeneous Scenario and Identifying the Potential Targets

In the majority of cases, HCC arises in the setting of chronic liver inflammation and cirrhosis, where a finely regulated immune environment becomes progressively dysregulated [61]. The liver is physiologically a tolerogenic organ, continuously exposed to gut-derived antigens, and this immune tolerance is maintained by specialized cells such as Kupffer cells and hepatic stellate cells, which promote regulatory T-cell activity and anti-inflammatory cytokine production [62,63,64].

In chronic liver disease, persistent inflammation leads to immune exhaustion, particularly affecting cytotoxic CD8^+^ T cells, which progressively lose antitumour activity and express inhibitory receptors such as programmed cell death protein-1 (PD-1) and cytotoxic T-lymphocyte-associated antigen-4 (CTLA-4) [65]. At the same time, tumour and stromal cells upregulate checkpoint ligands (e.g., PD-L1), further promoting immune escape and tumour progression [65]. Also, the tumour microenvironment (TME) in HCC is highly heterogeneous [66], encompassing a spectrum from inflamed tumours with active immune infiltration to immune-excluded or immune-desert phenotypes [67]. This heterogeneity has direct clinical implications, as it contributes to the variability in response to immune checkpoint inhibitors (ICIs) and influences recurrence risk after treatment, including LT.

From a clinical perspective, these insights support the rationale for immunotherapy-based strategies and for incorporating biological and immunological markers into patient selection. However, although immune profiling and molecular classification provide valuable prognostic information, their integration into routine clinical decision-making remains limited, and most evidence derives from retrospective studies or translational research rather than prospective clinical validation [68].

From a transplant oncology perspective, recognising TME heterogeneity reinforces the need to integrate molecular and immunological biomarkers into candidate selection. The traditional morphologic framework does not capture the dynamic interplay between tumour cells and the surrounding immune milieu. In this sense, a biologically informed approach that accounts for immune phenotype, stromal architecture, and clonal evolution may allow more accurate identification of patients with favourable disease biology despite borderline anatomical criteria [68].

### 3.2. Targeting Immune Checkpoints to Downstage the Tumor Burden: Overviewing the Role of Immune Checkpoint Inhibitors as Bridging Strategies to Liver Transplantation in HCC

#### 3.2.1. Rationale for Using ICIs in the HCC Pretransplant Setting

Recent therapeutic advances in HCC have incorporated first-line systemic therapies predominantly based on ICIs, specifically targeting the PD-1/PD-L1 axis and CTLA-4, in addition to molecules modulating the VEGF/VEGFR pathways.

Combination regimens—including atezolizumab plus bevacizumab, tremelimumab plus durvalumab, ipilimumab plus nivolumab, and camrelizumab plus rivoceranib—have consistently demonstrated superior overall survival compared with traditional tyrosine-kinase-based schemes, including sorafenib and lenvatinib, thereby redefining the standard of care [69].

Beyond their established role in advanced disease, these immune-based combinations are increasingly being explored in earlier stages of HCC management. In particular, these molecules are emerging as potential adjuncts or alternatives to conventional locoregional therapies, including TACE and TARE. In this context, the administration of ICIs in downstaging tumor burden or as bridging strategies aims to expand eligibility for LT. Within this context, these approaches are often conceptualized as part of a neoadjuvant therapeutic paradigm, reflecting a shift toward integrating systemic immunotherapy into multidisciplinary treatment algorithms for HCC [69].

The therapeutic synergy of CTLA-4 and PD-1/PD-L1 blockade in HCC is predicated on a dual-site reinvigoration of the anti-tumour immune response, addressing the unique tolerogenic nature of the liver. On one side, CTLA-4 inhibition primarily functions during the priming phase within the portal lymph nodes. In particular, by antagonising the competitive binding of CTLA-4 to B7 molecules (CD80/CD86, which also bind CD28), it facilitates the expansion of de novo T-cell clones and diminishes the suppressive influence of regulatory T-cells [70]. Conversely, PD-1/PD-L1 blockade operates at the effector phase within the TME, and this intervention disrupts the inhibitory signals delivered by HCC cells and Kupffer cells, thereby reversing T-cell exhaustion and restoring cytotoxic activity [71]. In the HIMALAYA study, this combined approach emerged as particularly efficient: while anti-CTLA-4 therapy increases the “breadth” of the immune repertoire by recruiting new effectors, anti-PD-1/PD-L1 therapy ensures these cells remain functionally active despite the immunosuppressive stroma characteristic of the cirrhotic liver [72].

As mentioned above, immunotherapy with a combination of PD-1/PD-L1 and VEGF inhibitors is also considered a gold standard for advanced HCC. The efficacy of the combination is not only due to their additive effects on tumour growth, but also to their synergistic effect, and the rationale lies in the sophisticated reversal of VEGF-mediated immunosuppression. Beyond its role in neo-angiogenesis, VEGF acts as a potent orchestrator of the TME by promoting the recruitment of myeloid-derived suppressor cells and regulatory T-cells, while simultaneously inhibiting dendritic cell maturation.

By antagonising the VEGF pathway, the intratumoral vasculature undergoes a “normalisation” phase, reducing interstitial fluid pressure and facilitating the efficient extravasation of tumour-infiltrating lymphocytes (TILs). This vascular recalibration, paired with the alleviation of cytokine-driven immune exclusion, creates a permissive environment for PD-1/PD-L1 blockade to effectively reinvigorate the anti-tumour T-cell response. Consequently, this combination therapy transforms the HCC stroma from an impenetrable, pro-oncogenic niche into an immunologically active site [30,73]. Overall, the progressive elucidation of these effects has strengthened the rationale for using ICI-based strategies in the pretransplant setting in routine clinical practice.

#### 3.2.2. Lights and Shadows of Using ICIs in the HCC Pretransplant Setting: State of the Art

In light of the abovementioned synergistic effects, ICIs have significantly changed the management of advanced HCC and are increasingly being explored as potential neoadjuvant strategies for downstaging or bridging selected patients to liver transplantation. In this sense, the rationale for using ICIs in the pretransplant setting—particularly PD-1/PD-L1 (atezolizumab, nivolumab, pembrolizumab, durvalumab) and CTLA-4 inhibitors (ipilimumab, tremelimumab)—rests on their capacity to achieve objective response rates [74]. Landmark combinations including atezolizumab–bevacizumab, durvalumab–tremelimumab, and nivolumab–ipilimumab have demonstrated remarkable radiologic response rates (up to 94%) and pathological response rates (35–88%), translating to one- and three-year post-transplant survival estimates of approximately 95% and 70–80%, respectively [74].

Current evidence-based integration of ICIs into transplant protocols emphasizes individualized, multidisciplinary decision-making rather than broad adoption. The IMbrave150 trial established atezolizumab–bevacizumab as first-line systemic therapy for advanced HCC, and extension of this combination into the neoadjuvant space has generated interesting proof-of-concept data [75].

Anyway, prospective randomized comparisons with TACE ± TARE or novel multimodal approaches (e.g., TACE combined with ICIs, as explored in EMERALD-1 and EMERALD-Y90 trials) remain ongoing and essential for determining optimal sequencing [75].

About this, comparative data between ICIs and TACE–TARE combinations suggest complementary rather than entirely competitive roles: a single-center analysis of combination therapy (TACE/TARE plus nivolumab–ipilimumab or atezolizumab–bevacizumab) versus locoregional therapy alone demonstrated no significant difference in post-transplant tumor recurrence or survival, though the combination cohort featured 3.5-fold higher tumor burden, implying that synergistic approaches may extend candidacy to patients with more advanced disease [76].

The neoadjuvant atezolizumab–bevacizumab combination exemplifies this paradigm: a prospective 17-patient cohort achieved 94% objective response, downstaging of 82% to within Milan criteria, and pathological response of 88%, with minimal grade 3–4 treatment-related adverse events, no severe post-transplant rejection, and no graft loss—outcomes that compare favorably to TACE or TARE [77].

The role of nivolumab–ipilimumab in the pretransplant setting remains limited, with data primarily from case series and single-arm studies suggesting feasibility but warranting larger prospective assessment given the elevated rejection rates associated with dual checkpoint blockade [78]. Therefore, safety considerations impose stringent requirements for ICI integration into transplant protocols.

Immune-related adverse events during neoadjuvant therapy—particularly hepatitis (occurring in 10–20% of treated patients)—demand rigorous monitoring and may necessitate temporary treatment interruption [79]. Moreover, post-transplant rejection following prior ICI exposure is not merely an academic concern. Concerning this, an analysis of 209 HCC patients receiving pre-transplant ICIs identified immune-related adverse events during the neoadjuvant phase as an independent predictor of subsequent allograft rejection (odds ratio -OR-: 9.170), alongside younger recipient age and washout <30 days [80]. In line with this, emerging evidence suggests that ICIs, particularly PD-L1 inhibitors, are associated with substantially higher post-transplant rejection rates (16–28%) compared to non-ICI cohorts (approximately 5–19%), with rejection onset typically occurring within the first three months after transplantation [81].

In this sense, indeed, the central challenge, which also distinguishes neoadjuvant immunotherapy from conventional locoregional approaches (TACE and TARE), concerns not merely efficacy but the critical issue of post-transplant rejection risk. While TACE and TARE remain essential bridging modalities—offering repeated treatability, cost-effectiveness, and well-established safety profiles—emerging evidence suggests that ICIs, particularly PD-L1 inhibitors, are associated with substantially higher post-transplant immune-related complications [74,81,82].

Comprehensively, this evidence highlights the relevance of considering transplant-specific risks of immunotherapy related to the administration of ICIs in the pretransplant setting. Moreover, despite the above-presented encouraging findings, currently available evidence should be interpreted with caution. Most reported outcomes derive from retrospective series, small single-center cohorts, and highly selected patient populations, which may overestimate treatment efficacy and underestimate toxicity. In addition, substantial clinical heterogeneity exists across studies concerning tumour stage, liver function, prior treatments, type of ICI, combination regimens, and washout intervals before transplantation. Standardized protocols for treatment duration, timing of LT after ICI exposure, and immunosuppressive management remain lacking. Therefore, although ICIs represent a promising strategy in selected candidates, their integration into transplant pathways still requires prospective validation and more uniform clinical frameworks.

### 3.3. HCC Immunotherapy-Related Transplant-Specific Risks: Mechanisms and Management

#### 3.3.1. Immune Memory Persistence After ICI Exposure

Although immunotherapy is increasingly being applied in HCC, including to help relocate patients initially excluded from the transplant list, this is not without risks related to the subsequent procedure. A paramount concern of the scientific community in this field is the prolonged pharmacodynamic effect and subsequent persistence of immune memory following ICI exposure. Evidence suggests that PD-1 receptor occupancy can endure for several months post-discontinuation, maintaining a state of heightened T-cell vigilance that bridges the pre-transplant treatment phase and the operative window regardless of inhibitor dose [83]. This persistence risks reprogramming the immune repertoire of the host towards a proinflammatory phenotype of human memory T lymphocytes, thereby lowering the threshold for alloantigen recognition. Consequently, upon liver graft implantation, the residual deregulated immune system may mount an aggressive, and often corticosteroid-resistant, rejection response. Integrating a mandatory wash-out period and monitoring T-cell receptor saturation may therefore be essential to mitigate the risk of hyperacute rejection in candidates previously exposed to immunotherapy [84,85].

#### 3.3.2. Allograft Rejection and Alloimmune Activation: Timing and Patient Selection

One of the central challenges of alloimmune activation in these patients lies in the fundamental disruption of the PD-1/PD-L1 axis, a pathway physiologically indispensable for maintaining the unique immunological privilege of the liver [86]. In the standard transplant setting, expression of PD-L1 on donor hepatocytes and liver sinusoidal endothelial cells contributes to peripheral T-cell regulation, attenuating alloreactive responses and promoting graft tolerance. Experimental models have demonstrated that PD-1/PD-L1 signalling limits alloimmune-mediated tissue injury, whereas its blockade accelerates rejection [87,88]. Pre-transplant exposure to immune checkpoint inhibitors effectively dismantles this regulatory safeguard. By blocking PD-1/PD-L1 signalling, ICIs may lower the activation threshold of recipient T cells, thereby enhancing recognition of donor major histocompatibility complex (MHC) antigens. Emerging clinical data suggest that this can result in a modified alloimmune response characterised by early and severe rejection episodes. Importantly, some case series and multicentre retrospective analyses report not merely an increased incidence of rejection, but a qualitative shift toward more aggressive and frequently steroid-refractory phenotypes [89,90].

Large cohort analyses have confirmed that liver transplant recipients previously exposed to ICIs may exhibit significantly higher rates of acute rejection and graft loss compared with patients managed with conventional locoregional therapies alone. Notably, rejections have been observed even when transplantation occurred several weeks after cessation of immunotherapy, suggesting a prolonged period of alloimmune vulnerability that may extend beyond the pharmacokinetic half-life of these agents [91].

In this regard, the optimal timing of liver transplantation following ICI exposure remains one of the most debated issues in transplant oncology. Although some ICIs such as nivolumab and pembrolizumab have relatively short pharmacokinetic half-lives (∼26 days) [92], their immunodynamic effects may persist substantially longer due to sustained T-cell activation and durable receptor occupancy [83,93]. This discrepancy between drug clearance and immune reprogramming underpins the concept that alloimmune vulnerability may extend beyond simple pharmacological washout, even though a very recent meta-analysis indicates that the use of ICIs for bridging or downstaging in the treatment of HCC before liver transplantation seems feasible, and a washout period spanning 1–3 months is considered reasonable to mitigate the risk of allograft rejection effectively [82].

On the other hand, multicentre studies showed that patients who receive a pre-LT ICI therapy with a TLAT (time interval between the last administration of ICI therapy and LT) shorter than 30 days have a much higher risk of allograft rejection than those with a TLAT longer than 30 days. In this study, in fact, multivariate logistic regression analysis showed that the TLAT ≥ 30 days was an independent protective factor for allograft rejection, and that allograft rejection was an independent risk factor for overall survival [94].

However, the concept of a rigid, universally applicable washout period may be overly simplistic. Instead, a risk-adapted strategy incorporating interval duration, depth of tumour response, immunological history, and, where available, biomarker assessment may provide a more rational framework for candidate selection within a multidisciplinary setting involving oncologists, hepatologists, and transplant surgeons [95]. In this context, although no universally accepted threshold exists, current evidence suggests that a minimum washout interval of 4–6 weeks should be considered, while longer intervals (up to 8–12 weeks) may be associated with a lower risk of allograft rejection, particularly in patients with prior immune-related adverse events [95].

At the same time, careful patient selection remains essential. Candidates most suitable for ICI-based downstaging strategies are those with preserved liver function, liver-confined disease, favourable biological features, and a documented radiological or biochemical response to therapy. Conversely, caution is warranted in patients with aggressive tumour biology, prior severe immune-related toxicity, or limited therapeutic alternatives in the event of post-transplant complications [95].

Definitely, peri-transplant management should be individualised and conducted within experienced transplant centres, with close monitoring for immune-related adverse events before transplantation and early signs of rejection after transplantation.

Overall, while ICIs represent a promising strategy to expand transplant eligibility, their use should currently remain restricted to carefully selected patients within structured clinical pathways. This cautious approach is further supported by the lack of standardised protocols and the limited level of evidence, which is largely derived from retrospective studies and expert consensus. Prospective registries and controlled trials are urgently needed to define optimal timing, selection criteria, and immunosuppressive strategies in this evolving field.

### 3.4. Peri-Transplant Immunosuppression Strategies in HCC in the Era of Immunotherapy

#### 3.4.1. Balancing Tumor Control and Graft Tolerance: CNIs and mTOR-Based Strategies

The optimal immunosuppressive strategy in patients undergoing liver transplantation after ICI exposure remains uncertain. In this context, the choice between calcineurin inhibitors (CNIs) and mechanistic target of rapamycin (mTOR) inhibitors carries not only implications for graft survival, but also for tumour recurrence and immune modulation.

Calcineurin inhibitors, including tacrolimus and cyclosporine, remain the backbone of post-liver transplant immunosuppression. By inhibiting NFAT-mediated transcription and IL-2 production, CNIs effectively suppress T-cell activation and reduce acute rejection rates. However, prolonged exposure to CNIs has been associated with increased oncogenic risk, potentially through impairment of immune surveillance and pro-angiogenic effects mediated by transforming growth factor-β and vascular endothelial growth factor pathways [43,96]. In the setting of HCC, higher cumulative CNI exposure has been correlated with increased recurrence rates following transplantation [97].

In contrast, mTOR inhibitors such as sirolimus and everolimus exert dual immunosuppressive and antiproliferative effects. By blocking the PI3K–AKT–mTOR pathway, these agents inhibit T-cell proliferation while simultaneously interfering with tumour cell growth, angiogenesis, and metabolic signalling [98].

Several retrospective analyses and prospective trials suggest that mTOR-based regimens may reduce HCC recurrence compared with CNI-dominant strategies, particularly in patients within standard transplant criteria [99]. The SiLVER trial, although not definitively conclusive for long-term benefit, demonstrated improved recurrence-free survival in certain subgroups receiving sirolimus-based therapy, especially in patients with AFP-evidence of higher tumour activity [100].

Physiologically, liver allograft acceptance depends on mechanisms of peripheral immune regulation, including PD-1/PD-L1 signalling, expansion of Tregs, and deletion or anergy of alloreactive T-cell clones [86,101]. Conversely, effective antitumour responses require reversal of T-cell exhaustion, increased cytotoxic activity, and sustained effector function, processes actively promoted by PD-1 blockade [102]. These pathways are mechanistically intertwined, such that amplification of one axis may destabilise the other, and clinical data increasingly illustrate this tension. Episodes of acute rejection following ICI exposure are often characterised by dense CD8^+^ infiltration and heightened inflammatory signatures, reflecting an immune milieu primed for cytotoxic activity rather than tolerance [89]. At the same time, excessive immunosuppression aimed at preventing rejection, particularly with high-dose calcineurin inhibitors, has been associated with increased HCC recurrence, likely through suppression of immune surveillance and pro-angiogenic signalling [97]. Thus, both insufficient and excessive immune modulation may adversely impact outcomes, and mTOR-based strategies have been proposed as a potential means of partially decoupling tumour control from alloimmune activation. In addition to their antiproliferative effects on tumour cells, mTOR inhibitors may preserve aspects of memory T-cell differentiation and exert less direct interference with immune checkpoint pathways compared with calcineurin inhibitors [99,103].

Although definitive prospective data are lacking, combination approaches involving reduced CNI exposure and early mTOR introduction represent a biologically plausible attempt to recalibrate this balance. In this setting, the goal is not maximal immune activation nor maximal suppression but controlled immune recalibration. Achieving this balance likely requires integration of tumour biology, timing of prior immunotherapy, immunological risk profiling, and dynamic monitoring of graft function. In this evolving landscape, balancing tumour control and graft tolerance emerges as a central pillar of precision transplant oncology.

#### 3.4.2. Emerging Immunomodulatory Approaches in Liver Transplantation in HCC

The growing intersection between immunotherapy and LT has stimulated interest in novel immunomodulatory strategies aimed at refining, rather than simply suppressing, alloimmune responses. In this evolving landscape, the objective is no longer broad immunosuppression, but selective immune recalibration capable of preserving graft tolerance while maintaining surveillance.

One emerging concept is the use of costimulatory pathway modulation. Blockade of CD28–CD80/86 signalling with agents such as belatacept has demonstrated efficacy in kidney transplantation by reducing calcineurin inhibitor exposure and preserving renal function [104]. Although experience in liver transplantation remains limited, selective costimulatory blockade theoretically offers a more targeted suppression of naïve T-cell activation without fully abolishing effector memory responses, potentially allowing a more nuanced immune balance in patients previously exposed to checkpoint inhibitors.

Adoptive and regulatory cell-based therapies represent another promising frontier.

Expansion or infusion of regulatory T cells has shown potential in early-phase transplant tolerance trials, with evidence suggesting that Treg-based strategies may promote operational tolerance and reduce dependence on pharmacological immunosuppression [105].

In the context of transplant oncology, such approaches could theoretically mitigate alloimmune activation without excessively impairing tumour-specific immunity, although clinical data remain preliminary.

Biomarker-guided immunosuppression represents another key development, and advances in immune profiling, including circulating donor-specific antibodies, T-cell receptor repertoire analysis, PD-1 expression kinetics, and gene-expression signatures associated with rejection, may allow dynamic risk stratification and personalised adjustment of immunosuppression intensity [106]. Such approaches align closely with the broader framework of precision transplant oncology, shifting from static protocols to adaptive immune monitoring.

Finally, peri-transplant desensitization and tailored induction regimens are being explored in high-risk patients previously treated with ICIs.

Agents such as anti-thymocyte globulin (ATG) or IL-2 receptor antagonists, such as basiliximab, may provide early control of alloimmune activation, though their impact on tumour recurrence requires careful evaluation [107].

Collectively, these emerging strategies reflect a paradigm shift from uniform immunosuppressive regimens to biologically informed, patient-specific immune modulation. As liver transplantation increasingly intersects with immunotherapy, the future of transplant oncology will depend on the ability to fine-tune immune equilibrium rather than simply suppress it.

## 4. Cholangiocarcinoma: From Experimental Protocols to Molecularly Guided Transplantation

### 4.1. Cholangiocarcinoma: An Umbrella Term Embracing Wide Clinical and Biological Heterogeneity

Representing the second most common primary liver cancer, CCA comprises a biologically and clinically heterogeneous spectrum of malignancy originating from the biliary epithelium and accounts for approximately 15% of all primary liver cancers worldwide [63]. Based on the anatomical location, CCA is currently classified into three distinct subtypes: intrahepatic (iCCA), perihilar (pCCA), and distal CCA (dCCA), each exhibiting distinct epidemiological trends, clinical behavior, therapeutic strategies, and prognostic trajectories [108,109].

Notably, iCCAs usually arise in the intrahepatic biliary tree, proximal to the second-order bile ducts, making it biologically closer to primary liver malignancies than to extrahepatic biliary tumors, and it comprises two distinct histological categories: small bile duct (SBD) and large bile duct (LBD) types [109]. Its incidence has increased significantly over recent decades, now representing 50–60% of all CCAs, particularly in high-income countries, likely reflecting evolving risk factors (e.g., MASLD), improved imaging techniques, and refined pathological classification [110].

Surgical resection remains the treatment of choice for localized disease, often requiring major hepatectomy, while advanced stages are primarily managed with systemic therapy, currently based on gemcitabine–cisplatin (GemCis) combinations with immunotherapy, ultimately configuring a scenario where the role of LT remains investigational in this setting [111].

Differently, pCCA develops in the right and/or left hepatic ducts and common hepatic duct, whereas the cystic duct delimits these tumours from dCCAs, which present distal to this in the common bile duct.

Notably, pCCA, accounting for approximately 25–35% of all cases, is frequently associated with primary sclerosing cholangitis (PSC) [112]. Surgical resection represents the standard curative option when technically feasible; however, many patients are diagnosed at an unresectable stage due to vascular involvement or biliary extension. In this setting, neoadjuvant chemoradiation followed by LT has emerged as a validated therapeutic strategy in carefully selected patients, fundamentally reshaping the historical contraindication of transplantation in this disease [111].

Beyond anatomical classification, comprehensive molecular profiling has revealed that CCA is a biologically heterogeneous disease with clinically relevant differences according to anatomical subtype [109]. In particular, intrahepatic CCA frequently harbours targetable alterations such as IDH1/2 mutations and FGFR2 fusions [113], whereas extrahepatic forms are more commonly associated with KRAS [114] and TP53 alterations [115].

The tumour immune microenvironment is similarly heterogeneous [116], ranging from inflamed phenotypes potentially sensitive to immunotherapy to immune-desert or stromal-rich tumours associated with resistance and poorer outcomes [117].

Collectively, these data underscore that CCA is not a single disease entity but a molecularly and immunologically heterogeneous continuum in which molecular and immune features are crucial in guiding prognostication and therapeutic decision-making. While these molecular insights are increasingly relevant for prognostic stratification and targeted therapy, their application in transplant selection remains largely investigational and requires prospective validation.

In line with this, the following sections, by consistently excluding the dCCA, since this entity does not primarily involve the hepatic parenchyma and requires pancreatic resection for oncologic clearance, will focus on the presentation of the most promising investigational protocols in the field of transplant oncology [108], opening the way for emerging strategies modulating the molecular targets in CCA.

### 4.2. Evolution of Liver Transplant Protocols for Cholangiocarcinoma: State of the Art

#### 4.2.1. Perihilar Cholangiocarcinoma: Neoadjuvant Protocols and Allocation Strategies

CCA is frequently diagnosed at advanced stages, thereby limiting surgical resectability due to vascular involvement, tumor location, or underlying liver disease. In this context, LT has progressively emerged as a potential curative strategy in highly selected patients [111].

Focusing on pCCA, the landmark publication of a specific protocol elaborated firstly by the University of Nebraska, later by the Mayo Clinic, and subsequently validated in multicenter cohorts, converted unresectable pCCA from a fatal condition into a potentially curable disease [118,119,120,121,122,123].

By demonstrating that neoadjuvant chemoradiation followed by LT could achieve 5-year overall survival rates exceeding 60% in carefully selected pCCA-affected patients, this protocol laid the foundation for modern transplant oncology [110,124].

The Mayo protocol combines external beam radiotherapy with radio-sensitizing fluoropyrimidine-based chemotherapy (typically, 5-fluorouracil or capecitabine), followed by intraluminal brachytherapy, maintenance capecitabine, staging laparoscopy (to rule out hidden metastases), and finally LT [125].

This multimodal approach extends beyond cytoreduction and local tumor control, effectively representing a biological selection strategy by excluding patients with rapidly progressive disease. Therefore, dropout during the waiting period (ranging between 30 and 40%) emerges as a critical biological sign rather than merely a negative outcome [122,126].

In this context, considering that patient selection represents the most important determinant of post-LT outcomes, the United Network for Organ Sharing (UNOS) provided a standardized pathway for carefully selected patients, relying on the following criteria: unresectable but non-metastatic pCCA ≤3 cm, absence of lymph node involvement or distant metastases, no prior transperitoneal biopsy to avoid the risk of tumor seeding, completion of standardized neoadjuvant protocol, stable disease without progression during the waiting period [122,126]. Although comprehensive analysis revealed that only about 5% of all patients presenting with pCCA may be eligible for LT, in patients meeting the abovementioned criteria, 5-year overall survival rates of approximately 60–70% have been consistently reported across multiple centers, approaching outcomes observed for HCC within accepted transplant criteria [122,126].

Supporting these data, a meta-analysis including 20 studies and 428 patients demonstrated that neoadjuvant chemoradiation followed by LT achieved 1-, 3-, and 5-year overall survival rates of 82.8%, 65.5%, and 65.1%, respectively, substantially superior to transplantation alone (71.2%, 48.0%, and 31.6%), and consistent with the results reported from The Irish National Liver Transplant Programme [127,128].

Interestingly, a comprehensive sequential series, spanning from 1993 to 2018 and encompassing 211 patients who underwent LT, has revealed an overall survival after transplantation for patients with PSC-associated pCCA (*n* = 138) of 92%, 76% and 70% at 1, 5 and 10 years, respectively, compared to 90%, 58% and 49% for patients with sporadic or de novo pCCA (*n* = 73) [129]. More recently, allocation policy refinements have further improved access to transplantation [130]. Shaikh et al. demonstrated that implementation of the Median MELD at Transplant minus 3 (MMaT-3) policy, by assigning candidates a MELD score three points below the local median MELD at transplant, significantly increased the probability of LT at one year (56.0% vs. 73.6%) and reduced three-year waitlist mortality (26.6% vs. 17.6%), without compromising post-procedural survival [130]. These findings highlight the critical role of allocation systems in ensuring equitable access while maintaining excellent oncologic outcomes.

Living donor liver transplantation (LDLT) represents an additional strategy to overcome organ shortage and reduce dropout. Consistently, Japanese prospective experiences have reported excellent short-term survival following LDLT for unresectable pCCA after neoadjuvant therapy, although vascular complications such as hepatic artery thrombosis remain relevant technical concerns [131]. Notably, encouraging results with different neoadjuvant protocols, such as CisGem with or without radiotherapy, have emerged recently, ultimately aiming to circumvent the perioperative morbidity associated with neoadjuvant radiation [124,132,133,134].

#### 4.2.2. Neoadjuvant Strategies and Liver Transplantation in Intrahepatic Cholangiocarcinoma: Current Evidence and Ongoing Trials

While neoadjuvant chemoradiation is well established for pCCA, its role in iCCA remains investigational, and orthotopic liver transplantation (OLT) in this setting should be considered only in clinical trials, due to reported high recurrence rates and poor survival. However, a turning point occurred with the international retrospective study by Sapisochin et al. with the iCCA International Consortium [135].

This cornerstone research demonstrated that patients transplanted for other indications, and later incidentally diagnosed with “very early” iCCA, particularly in the setting of cirrhosis, achieved promising survival rates, highlighting tumor size as a critical determinant of recurrence risk, particularly in the case of tumours exceeding 2 cm [135]. These findings reintroduced LT as a potential option in highly selected patients with very early iCCA and supported the need for the construction of prospective trials (ClinicalTrials.gov Identifier: NCT04556214; ClinicalTrials.gov Identifier: NCT02878473).

With this purpose, the TESLA Trial (ClinicalTrials.gov Identifier: NCT04556214), sponsored by Oslo University Hospital, represents the first prospective effort to evaluate LT in patients with unresectable iCCA demonstrating disease stability after at least six months of systemic therapy (gemcitabine-based chemotherapy regimen) [136].

More recently, additional Italian prospective protocols have been proposed to further refine selection criteria for liver LT in this target population through biologically driven approaches. The iCOLA trial (ClinicalTrials.gov Identifier: NCT06862934) is evaluating a multimodal downstaging strategy combining systemic chemotherapy with or without immunotherapy and transarterial radioembolization, followed by transplantation in patients demonstrating sustained disease control [109].

In parallel, the LIRICA study (ClinicalTrials.gov Identifier: NCT06098547) integrates translational biomarkers and advanced imaging into the selection algorithm, aiming to identify molecular and radiological predictors of favorable tumor biology and optimize candidate stratification beyond conventional morphological criteria [109]. Preliminary results by an intention-to-treat analysis from Maspero et al. revealed the efficacy of combined systemic chemotherapy and radioembolization (SYS-TARE) to downstage unresectable iCCA before LT, with a subset of patients achieving long-term post-transplant disease-free survival [137].

However, these data remain limited by small sample sizes and require prospective validation. Innovative approaches, including staged hepatectomy with auxiliary LDLT, as investigated in the LIVINCA trial, further reflect attempts to expand transplant eligibility in this traditionally contraindicated population [138].

Altogether, these efforts reflect the broader trend toward individualized, response-guided selection of transplant candidates beyond morphological criteria, as well as the absolute need to consider the biological heterogeneity of this cancer to develop molecular-based strategies to include in protocols that can concretely and individually expand the candidacy to CCA.

### 4.3. The “Biological Downstage”: Modulating Molecular Targets to Expand the Frontiers of Transplant Candidacy and Curative Outcomes in CCA

The expanding molecular stratification has enthusiastically and simultaneously opened the door to the development of new targeted agents, creating unprecedented opportunities and reshaping the CCA-therapeutic landscape.

The phase III ClarIDHy trial demonstrated that the IDH1 inhibitor ivosidenib significantly improved progression-free survival compared with placebo in previously treated IDH1-mutant CCA, leading to regulatory approval by both the FDA and EMA, as well as the FGFR inhibitors pemigatinib and futibatinib in FGFR2-rearranged CCA [72,139].

In parallel, additional targeted strategies are emerging: HER2-directed therapies, combined B-Raf proto-oncogene, serine/threonine kinase (BRAF)/MEK inhibition, and investigational approaches targeting MDM2 amplification, KRAS^G12C mutations, Claudin 18.2 expression, homologous recombination deficiency (BRCA1/2, PALB2), and MTAP deletions [109,140].

In light of this rapidly evolving pharmacologic landscape, it is both conceivable and stimulating to explore whether targeted therapies may extend beyond the approved advanced iCCA-setting and be incorporated into neoadjuvant pre-transplant strategies to achieve biological downstaging and enable access to potentially curative therapeutic strategies. In this context, while pre-operative or pre-transplant protocols remain investigational, early clinical experiences, although limited, suggest that molecularly selected patients may achieve meaningful tumor regression, potentially expanding resectability or transplant candidacy [137,141].

Notably, a recent case report from Byrne et al. described a successful biological downstaging of an FGFR2-mutated iCCA-affected patient with pemigatinib (a FGFR2 inhibitor), leading to sustained radiologic response after 2 months and subsequently eligible to LDLT after 6 months, with no evidence of recurrence at short-term follow-up [141]. Consistently, in a study by Maspero et al., a FGFR2-rearranged iCCA-affected patient who received pemigatinib as second-line systemic therapy before LT demonstrated remarkable pathological responses, with <5% viable tumor cells identified in the explanted liver [137].

Among prospective trials exploring targeted agents within neoadjuvant frameworks, the OPT-IC trial (ClinicalTrials.gov Identifier: NCT03579771) was designed to evaluate the efficacy of an FGFR-inhibitor in addition to a gemcitabine-based chemotherapy in patients with FGFR2-rearranged iCCA prior to surgery.

Although primarily conceived for surgical resection rather than LT, it represents one of the first structured attempts to integrate molecularly guided therapy into a preoperative downstaging framework and may inform future transplant-oriented protocols. In this context, neoadjuvant targeted therapy could serve not only as a cytoreductive strategy but also as a dynamic test of tumor biology: patients who achieve prolonged radiologic and metabolic stability under genotype-matched therapy may represent a subgroup with lower metastatic competence and may derive long-term benefit from transplantation; conversely, early progression despite targeted inhibition may signal aggressive clonal heterogeneity and high post-LT recurrence risk [109].

Moreover, beyond the direct effects exerted by targeted molecules, experimental models suggest that oncogenic pathways such as FGFR and IDH1 signaling interact bidirectionally with stromal programs, indicating that targeted inhibition may affect the environmental compartment [109]. FGFR inhibition, for instance, has been associated with modulation of downstream MAPK and PI3K pathways, potentially reducing proliferative signaling and altering cytokine networks within the TME [142,143]. Similarly, pharmacologic IDH1 inhibition has been shown in preclinical settings to partially restore immune signaling and differentiation programs, suggesting that targeted therapy may have immunomodulatory consequences [144,145].

In this scenario, considering the abundance of cancer-associated fibroblast (CAF) subsets within the TME, which modulate extracellular matrix, angiogenesis, immune suppression, and tumour-stroma interactions, it appears plausible to consider the development of novel therapeutic approaches directly targeting CAFs or CAF-mediated pathways [109].

However, prospective evidence will be essential to confirm and expand the currently preliminary, albeit encouraging, data available in the literature, further defining optimal duration of therapy, integration with systemic chemotherapy or locoregional treatments, assessment of minimal residual disease, and the potential impact of prolonged targeted exposure on post-transplant immunosuppression and recurrence dynamics.

Table 1 summarizes the state of the art for LT strategies in the management of CCA (Table 1).

## 5. Colorectal Liver Metastases: Revisiting Transplantation for Secondary Liver Cancer

### 5.1. Biological Features of Colorectal Cancer: Understanding the Molecular Bases to Identify the Determinants of Favorable Outcomes and Guide LT-Candidacy Patient Selection

#### 5.1.1. Colorectal Cancer as a Heterogeneous Entity: Overviewing the Concepts of “Clonal Indolence”, Low Metastatic Fitness, and Organotropism

Metastatic colorectal cancer is a heterogeneous entity, representing a spectrum of clonal dynamics ranging from rapidly progressive, genomically unstable disease to more slowly evolving, liver-confined oligometastatic phenotypes [146,147]. Genomic studies have demonstrated that metastatic dissemination in colorectal cancer often follows a branched evolutionary pattern, in which dominant clones seed distant sites while maintaining varying degrees of genetic heterogeneity relative to the primary tumour [49]. Importantly, a subset of patients exhibits limited clonal diversification and relatively stable genomic architecture, features associated with slower tumour growth and improved response to systemic therapy [148]. In these cases, the liver may serve as the predominant, and sometimes exclusive, metastatic reservoir for prolonged periods, supporting the biological rationale for radical locoregional strategies and even transplantation, with molecular profiling having further refined this concept. About this, indeed, tumours harbouring KRAS or BRAF mutations are consistently associated with more aggressive behaviour, early recurrence, and inferior survival following both resection and transplantation [149,150]. Conversely, KRAS wild-type status may serve as a surrogate marker of biologically indolent disease [29]. In the aforementioned SECA studies, favourable molecular and clinical profiles correlated strongly with prolonged post-transplant survival, reinforcing the principle that tumour biology drives outcome rather than tumour burden alone [47].

Beyond clonal indolence, another critical biological determinant underpinning favorable outcomes after LT for CRLM is the concept of low metastatic fitness and restricted organotropism. Interestingly, indeed, not all metastatic clones possess equal systemic dissemination capacity [151].

Rather, metastatic competence reflects a highly selective evolutionary process requiring tumour cells to acquire specific molecular adaptations enabling survival in circulation, immune evasion, and colonisation of distant niches [148,151].

Experimental and translational studies have demonstrated that metastatic spread is not a stochastic event, but a biologically constrained phenomenon governed by tumour–host interactions and microenvironmental compatibility, according to the classic “seed and soil” paradigm first articulated by Paget in 1889 [152]. In colorectal cancer, the liver represents the predominant metastatic site due to anatomical factors, portal circulation, and molecular factors favouring hepatic colonisation [153]. However, only a subset of tumours remains persistently confined to the liver, suggesting limited competence for further systemic dissemination. Low metastatic fitness may manifest clinically as prolonged liver-only disease despite adequate observation time and systemic therapy exposure. Such tumours often demonstrate sustained response or stability under chemotherapy, absence of extrahepatic progression during prolonged follow-up, and relatively low circulating tumour DNA (ctDNA) levels, all indirect indicators of constrained systemic spread potential [154,155]. From an evolutionary standpoint, these clones may lack additional genetic or epigenetic alterations required for successful colonisation of other organs, thereby exhibiting restricted organotropism, and this stratification is also supported by molecular determinants. As partially mentioned previously, RAS and BRAF wild-type status profiles have been associated with less aggressive metastatic behaviour [149]. Conversely, tumours harbouring BRAF mutations demonstrate higher metastatic plasticity and poorer post-resection outcomes, raising concerns regarding transplant candidacy in such cases [147]. Importantly, the Oslo experience suggested that recurrence after transplantation frequently occurs in the lungs rather than diffusely or systemically, and that pulmonary recurrences often follow a relatively indolent course amenable to resection [47].

This pattern reinforces the notion that certain CRLM phenotypes exhibit limited metastatic fitness rather than uncontrolled systemic dissemination.

In selected patients, LT may act as a definitive strategy to eradicate the dominant hepatic reservoir, while residual metastatic potential remains biologically constrained and clinically manageable.

Taken together, the integration of metastatic fitness and organotropism into transplant selection moves the field beyond static tumour burden metrics. Instead, it embraces a dynamic biological model in which transplantation is reserved for patients whose tumour evolutionary trajectory suggests confinement rather than systemic inevitability. Identifying and validating biomarkers of low metastatic competence will be central to safely expanding this indication.

#### 5.1.2. Exploring the Central Role of Immune Surveillance in Colorectal Cancer

An additional, often understated determinant of favorable outcomes in this setting of patients remains the role of host immune surveillance. The capacity of the immune system to recognize and constrain residual tumour cells plays a fundamental role in shaping metastatic progression, even in advanced disease settings [156]. The concept of cancer immunoediting describes a dynamic equilibrium between tumour cells and the host immune response, comprising elimination, equilibrium, and escape phases, “the three Es” [157].

In certain patients with liver-confined CRLM, prolonged disease stability under systemic therapy may reflect a state of partial immune containment, in which cytotoxic T lymphocytes and innate immune mechanisms limit systemic dissemination without achieving complete tumour eradication. This equilibrium state may help explain why some patients exhibit durable liver-only disease despite extensive tumour burden, and the immune contexture of colorectal cancer has been shown to correlate strongly with prognosis. High densities of TILs, particularly CD8^+^ cytotoxic T cells, are associated with improved survival in both primary colorectal cancer and metastatic lesions [158].

The Immunoscore framework has further validated the prognostic significance of adaptive immune infiltration, demonstrating that immune parameters can outperform traditional TNM staging in predicting outcomes [159].

Although most data derive from primary tumours, similar principles appear to apply to metastatic sites, including the liver. Importantly, in the transplant setting, pre-existing immune surveillance may influence post-transplant recurrence patterns. Tumours that have evolved under sustained immune pressure may exhibit lower metastatic plasticity or reduced capacity for rapid systemic expansion. Conversely, cancers characterised by early immune escape mechanisms, such as defective antigen presentation or immunosuppressive cytokine signalling, may be less suitable for transplantation due to higher recurrence risk under post-transplant immunosuppression [160].

Thus, beyond genomic and clinical selection criteria, the host–tumour immune interaction represents a critical biological filter. Incorporating immune biomarkers such as TIL density, immune gene signatures, or circulating immune profiles into transplant evaluation frameworks may enhance the precision of candidate selection. In this perspective, favourable transplant outcomes may not only reflect tumour-intrinsic indolence but also the continued restraining influence of an intact and competent immune surveillance system.

### 5.2. Integrating Molecular and Immunologic Stratification as a Crucial Step for Guiding Patient Selection and Expanding Liver Transplantation Candidacy

#### 5.2.1. Determining RAS/BRAF Mutation Status and Tumor Sidedness in Colorectal Cancer

The incorporation of molecular profiling into candidate selection represents one of the most important advances in transplant oncology for CRLM. Among molecular determinants, RAS and BRAF mutational status have emerged as robust prognostic biomarkers across surgical and systemic treatment settings and are increasingly relevant when considering liver transplantation [29,49].

KRAS and NRAS mutations occur in approximately 40–50% of metastatic colorectal cancers and are consistently associated with inferior overall survival, increased recurrence rates, and reduced responsiveness to anti-EGFR therapies [49].

In the context of liver resection for CRLM, RAS mutations correlate with shorter recurrence-free survival and higher risk of extrahepatic spread, suggesting a biologically more aggressive phenotype. Importantly, similar trends have been observed in transplant cohorts, where RAS-mutant tumours demonstrate earlier recurrence and less favourable post-transplant outcomes [47].

BRAF mutations, particularly the V600E variant, confer an even more adverse prognosis: although present in a smaller proportion of patients (approximately 8–10%), BRAF-mutant metastatic colorectal cancer is associated with rapid progression, poor response to conventional chemotherapy, and limited long-term survival [161]. Given this aggressive biological behaviour, most contemporary transplant programs consider BRAF-mutant CRLM a relative or absolute contraindication outside experimental protocols.

In this scenario, tumour sidedness has also emerged as a clinically relevant surrogate of underlying tumour biology. Right-sided primary tumours, originating from the caecum to the transverse colon, are more frequently associated with BRAF mutations, microsatellite instability (MSI), CpG island methylator phenotype (CIMP), and consensus molecular subtype CMS1 or CMS4, features linked to aggressive behaviour and inferior survival in the metastatic setting [147,162]. Conversely, left-sided tumours more commonly harbour RAS wild-type profiles and exhibit better response to anti-EGFR therapy, translating into improved survival outcomes [163]. Large clinical analyses have confirmed that primary tumour location independently predicts survival in metastatic colorectal cancer [164].

#### 5.2.2. Beyond Genetic Mutations and Tumor Sidedness: Time for Consensus Molecular Subtypes (CMS)

While all these individual mutations provide important prognostic information, they capture only part of the biological heterogeneity of colorectal cancer, and a more comprehensive framework is offered by the Consensus Molecular Subtypes (CMS) classification, which integrates transcriptomic, genomic, and microenvironmental features into four biologically distinct categories [162].

This classification identifies four major subtypes: CMS1 (microsatellite instability immune), CMS2 (Canonical), CMS3 (Metabolic), and CMS4 (Mesenchymal) [147,162]. Each subtype reflects a distinct tumour–host interaction pattern and carries specific prognostic implications in metastatic disease.

CMS1 tumours are characterised by microsatellite instability (MSI), high tumour mutational burden, and prominent immune infiltration. Although this subtype often demonstrates sensitivity to immune checkpoint inhibition, it may also exhibit early relapse patterns in metastatic settings and is frequently associated with right-sided primaries and BRAF mutations [165]. In the transplant context, the high immunogenicity of CMS1 tumours poses a complex paradox: while immune responsiveness may favour systemic control, the requirement for post-transplant immunosuppression could theoretically attenuate this endogenous antitumour surveillance.

CMS2, the “canonical” subtype, is marked by chromosomal instability, activation of WNT and MYC pathways, and relatively epithelial differentiation. This subtype is more frequently left-sided, RAS wild-type, and generally associated with more favourable survival compared to CMS4 in the metastatic setting [166]. Patients within this group may represent biologically suitable candidates for transplantation when other clinical criteria are met.

CMS3 tumours display metabolic dysregulation and mixed molecular features, with intermediate prognostic behaviour. Although less extensively studied in metastatic cohorts, CMS3 does not appear to confer the aggressive stromal-driven phenotype observed in CMS4.

CMS4, the mesenchymal subtype, is characterised by stromal infiltration, TGF-β activation, angiogenesis, and prominent epithelial–mesenchymal transition (EMT) signatures [167]. Clinically, CMS4 is associated with worse overall survival, higher rates of recurrence, and increased metastatic plasticity. The strong stromal and pro-angiogenic signalling suggests enhanced capacity for systemic dissemination and immune evasion, raising concerns regarding suitability for transplantation, particularly under chronic immunosuppression.

Importantly, CMS classification also reflects the immune landscape of the tumour microenvironment. CMS1 tumours are typically “immune hot,” CMS4 often displays immunosuppressive stromal features, and CMS2/CMS3 are relatively immune intermediate. This heterogeneity underscores the potential value of integrating transcriptomic immune signatures into transplant candidate evaluation, particularly in the era of immunotherapy and precision immunosuppression.

Although CMS stratification is not yet routinely implemented in transplant selection algorithms, it provides a biologically coherent framework that moves beyond single-gene mutations. Incorporating CMS profiling into prospective studies may help identify subsets of CRLM patients whose tumour–microenvironment interactions are compatible with durable post-transplant disease control.

#### 5.2.3. Determining Immune Infiltrates and Checkpoint Expression in CRLM

Beyond genomic alterations and transcriptomic subtyping, the density, composition, and functional state of immune infiltrates within CRLM represent critical determinants of tumour behaviour and may have direct implications for transplant candidacy.

The prognostic significance of tumour-infiltrating lymphocytes (TILs) in colorectal cancer is well established. High densities of CD8^+^ cytotoxic T cells and memory T cells correlate with improved overall and disease-free survival in primary colorectal tumours [158,159]. Emerging evidence suggests that similar principles apply to metastatic deposits, including liver metastases, where a robust intratumoral immune infiltrate is associated with better post-resection outcomes [168]. This supports the notion that immune-active metastases may reflect partially contained disease, potentially compatible with radical strategies such as transplantation in carefully selected patients. However, immune infiltration alone does not equate to effective antitumour immunity, and the functional state of these infiltrating cells is equally relevant. Chronic antigen exposure within the tumour microenvironment frequently induces T-cell exhaustion, characterised by upregulation of inhibitory checkpoint molecules such as PD-1, CTLA-4, TIM-3, and LAG-3 [169].

In colorectal cancer, PD-L1 expression can be detected both on tumour cells and tumour-associated immune cells, although its prevalence and prognostic significance vary across molecular subtypes [170]. Checkpoint expression has complex implications in the transplant setting. On one hand, tumours with pre-existing immune infiltration and checkpoint upregulation may be biologically “visible” to the immune system, suggesting that endogenous immune control has been operative. On the other hand, post-transplant immunosuppression may blunt this residual immune containment, potentially facilitating tumour outgrowth. Furthermore, prior exposure to immune checkpoint inhibitors, increasingly common in MSI-high tumours, may alter alloimmune responses and impact graft tolerance, adding another layer of complexity [84]. From a stratification perspective, integrating immune infiltrate density, immune gene expression signatures, and checkpoint expression profiles may enhance risk prediction beyond conventional molecular markers. An “immune-inflamed” metastasis with preserved cytotoxic infiltration but limited systemic dissemination might represent a different biological entity from an immune-desert or stromal-dominated metastasis characterised by immune exclusion. Such distinctions may prove particularly relevant in anticipating recurrence dynamics under chronic post-transplant immunosuppression.

Ultimately, immune infiltrates and checkpoint expression provide a functional readout of tumour–host interaction. Incorporating these parameters into transplant evaluation frameworks aligns with the broader shift toward precision transplant oncology, in which candidacy is defined not only by tumour genetics but also by the immune ecology in which the tumour evolves.

### 5.3. Liver Transplant in Metastatic Colorectal Cancer: Clinical Evidence and Emerging Models

#### 5.3.1. Overviewing the Prospective and Retrospective Transplant Series: State of the Art

As previously mentioned, the Norwegian SECA Programme represented the turning point that led to the modern reconsideration of liver transplantation for CRLMs. In the SECA-I trial, 21 patients with unresectable liver-only CRLM underwent liver transplantation after modern chemotherapy.

Despite a high recurrence rate of approximately 90%, the 5-year overall survival reached 60%, significantly exceeding survival expected with chemotherapy alone in this setting [46]. Notably, many recurrences were slow-growing pulmonary metastases amenable to resection, challenging the traditional assumption that recurrence after transplantation necessarily implied rapid systemic failure.

The subsequent SECA-II study applied stricter selection criteria, including sustained response to chemotherapy, lower tumour burden, limited CEA levels, and longer interval from primary tumour resection. Under these refined conditions, 5-year overall survival increased to approximately 83%, approaching outcomes observed in established transplant indications [47]. Importantly, disease-free survival also improved compared to SECA-I, underscoring the impact of biological selection. Parallel retrospective analyses from European centres have supported these findings. Toso et al. reported that carefully selected patients transplanted for CRLM achieved encouraging long-term survival, particularly in the absence of adverse molecular features [48]. More recently, registry-based and multicentre analyses have begun to confirm that survival after transplantation in highly selected CRLM patients may surpass outcomes achieved with systemic therapy alone and, in some cases, approach those of extended liver resection strategies [45].

Comparative modelling studies further reinforce this paradigm shift. When matched against contemporary chemotherapy cohorts, transplant recipients demonstrate superior long-term survival despite early recurrence patterns. This suggests that transplantation may alter the natural history of liver-confined metastatic disease by removing the dominant tumour reservoir, even if systemic microscopic disease persists. Importantly, these data must be interpreted within the context of strict selection frameworks and controlled clinical environments. Outcomes cannot be extrapolated to unselected metastatic populations. Rather, the emerging evidence supports a model in which transplantation may offer a survival advantage in a biologically defined subset characterised by liver-only disease, favourable molecular profile, sustained response to systemic therapy, and absence of rapid systemic progression. Collectively, prospective and retrospective series provide proof-of-concept that CRLM is not an absolute contraindication to LT. Instead, they lay the clinical foundation for structured selection algorithms and prospective validation trials aimed at defining reproducible criteria for safe expansion of transplant eligibility.

Conclusively, the accumulation of clinical data from prospective and retrospective transplant series, combined with evolving insights into tumour biology, highlights a growing consensus: eligibility for LT in CRLM should be defined not solely by anatomical resectability but by integrated biological risk profiles. As described before, modern evidence reveals that a subset of patients may derive meaningful survival benefit from complete hepatic replacement, and prospective studies, such as SECA-I and SECA-II, demonstrated survival rates that approach those seen in standard transplant indications when selection is rigorous. Emerging selection models increasingly incorporate dynamic metrics such as tumour response kinetics, circulating biomarkers, including CEA and ctDNA, and composite risk scores that reflect both tumour biology and host factors. For example, time elapsed since initial diagnosis and stability under systemic therapy serve as surrogate biological stress tests for metastatic fitness [154]. Similarly, molecular stratification, including consensus molecular subtypes and immune phenotypes, offers additional granularity beyond traditional clinical staging [162]. The clinical narrative is transitioning from the reflexive exclusion of CRLM towards the identification of biologically favourable phenotypes. This entails seeking out disease states where constrained evolutionary trajectories and a lack of systemic dissemination suggest that the malignancy can be effectively managed, even within the permissive immune environment required for transplantation. Codifying these biological insights into robust, reproducible selection tools will be pivotal in the context of expanding transplant indications without compromising the ethical principles of equitable organ allocation.

Integrating these biological insights allows clinicians to transcend the view of LT as a reactive salvage procedure. Instead, this approach emerges as a proactive, targeted intervention for select patients, representing a significant departure from the conventional boundaries of surgical oncology in the metastatic setting.

#### 5.3.2. Beyond Resectability: Positioning Liver Transplantation Among Surgical Strategies for Colorectal Liver Metastases

A critical question in evaluating liver transplantation for CRLM remains how it compares with alternative surgical strategies in patients with extensive but liver-confined disease. Extended hepatectomy, two-stage hepatectomy, portal vein embolisation (PVE), and associating liver partition and portal vein ligation for staged hepatectomy (ALPPS) have significantly expanded resectability boundaries over the past two decades [50,171]. In carefully selected patients, these approaches can achieve 5-year overall survival rates approaching 40–50%, particularly when complete (R0) resection is obtained; however, recurrence rates remain high, reflecting the persistence of microscopic intrahepatic disease or early systemic spread [172].

Transplantation differs conceptually from resection in that it removes the entire hepatic parenchyma, potentially eradicating occult micrometastatic foci not detectable on imaging. This theoretical advantage is particularly relevant in patients with extensive bilobar disease, where multiple resections or staged procedures would be required. In the SECA experience, overall survival following transplantation compared favourably with historical resection cohorts of patients with similarly high tumour burden, despite a substantial rate of post-transplant recurrence [46].

Another emerging comparator is the RAPID concept (Resection and Partial Liver segment 2/3 Transplantation with Delayed total hepatectomy), which combines auxiliary partial liver transplantation with staged hepatectomy to overcome graft scarcity and facilitate regeneration before complete hepatectomy, an approach originally described by Line and colleagues as a potential strategy to broaden transplant applicability in non-resectable liver metastases [173].

Early feasibility data suggest that such hybrid approaches may broaden therapeutic options for patients otherwise deemed unresectable, although long-term oncologic outcomes remain under investigation. Importantly, transplantation should not be framed as a competitor to conventional resection in resectable disease. For patients amenable to R0 hepatectomy with adequate future liver remnant, resection remains the standard of care.

Rather, transplantation occupies a niche between resectable and systemically disseminated disease, targeting patients with technically unresectable yet biologically favourable liver-only metastases. Thus, the comparison with alternative surgical strategies underscores a shift from a purely anatomical definition of resectability to a biologically informed decision model. The key question is no longer whether disease can be technically removed, but whether complete hepatic replacement offers a survival advantage in patients whose tumour biology supports durable control.

In summary, emerging clinical evidence supports liver transplantation as a viable therapeutic option in highly selected patients with CRLM, where biological behavior rather than anatomical resectability drives outcomes. Comparative analyses with advanced surgical strategies highlight that transplantation may offer a survival advantage in patients with liver-confined, biologically favorable disease. Future efforts should focus on refining biologically driven selection models to safely integrate transplantation into the therapeutic algorithm of metastatic colorectal cancer.

**Figure 2:**
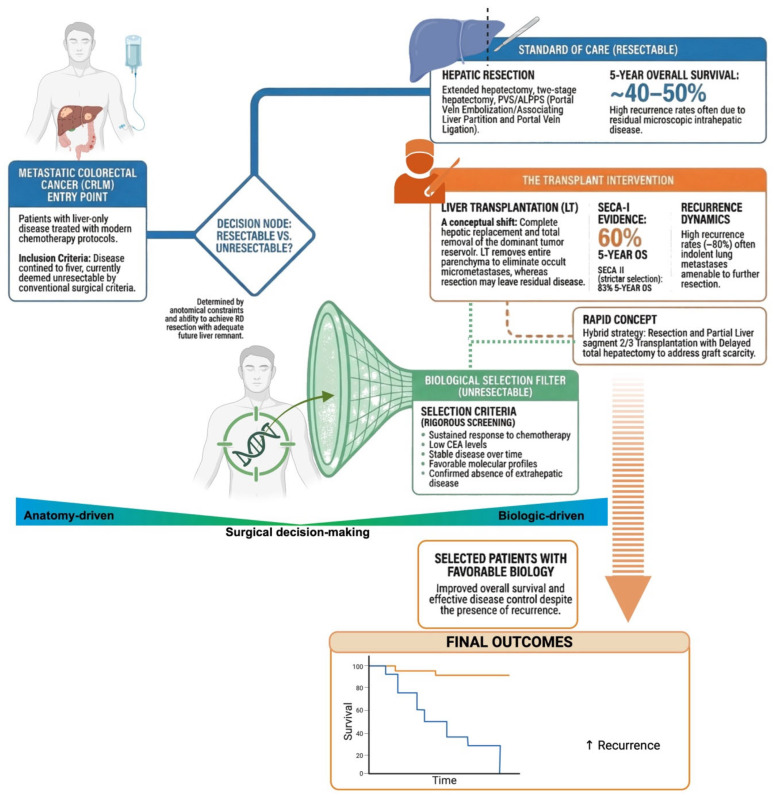
From anatomy to biology: the clinical positioning of Transplantation in Colorectal Liver Metastases. OS: overall survival; CEA: carcinoembryonic antigen; LT: liver transplant; SECA: Secondary Cancer study.

Figure 2 summarizes the potential applicability of LT in managing CRLM in the era of paradigm shift from “anatomy-driven” to “biology-driven” decision making (Figure 2).

## 6. From Bench to Bedside: Predicting the Outcomes After Liver Transplantation in the Era of “Personalized” Liver Transplant Oncology

### 6.1. Exploring the Biological Determinants of Oncologic Outcomes After Liver Transplantation

#### 6.1.1. Tumor Biology Is the Main Driver of Recurrence: Mechanisms and Targeting Strategies

Although LT represents a potentially curative option for selected patients with PLCs and secondary liver malignancies, post-transplant tumor recurrence remains a non-negligible limitation and varies widely according to tumor type and intrinsic biology [174,175,176]. Despite strict selection criteria, recurrence rates range from approximately 8–20% in HCC to nearly 30–50% in CRLM, and may exceed 50% in CCA, highlighting critical limitations in current selection and recurrence prediction strategies and driving the growing focus on transplantation oncology as a biologically driven, precision-based discipline [174,175,176]. In line with this, emerging evidence suggests that oncologic outcomes after LT are primarily determined by the underlying molecular profile and tumor biology rather than by morphological burden alone [175].

Consistently, beyond tumor differentiation and (micro-)vascular invasion, the most robust traditional biological predictors of recurrence and poor survival, a rapidly expanding array of molecular determinants is fundamentally redefining tumor classification, biological staging, and therapeutic decision-making in hepatobiliary malignancies [175,177].

Among these, clonal heterogeneity, a concept widely documented in HCC, is emerging as a crucial determinant of tumor behavior, enabling different nodules or even areas within the same tumor to display divergent genomic and proteomic profiles that influence metastatic potential and post-transplant behavior [177,178]. Additionally, EMT programs promote metastatic competence, leading to tumor cell migration, invasion, and vascular dissemination. Key EMT-related mediators, including MMP1 (Matrix Metalloproteinase 1) and SPP1 (Osteopontin), have been consistently associated with vascular invasion, early recurrence, and reduced recurrence-free survival in HCC, while activation of thrombin-related pathways further facilitates tumor cell adhesion to the endothelium and extracellular matrix, thereby facilitating distant spread [179].

In line with this, post-transplant outcomes are fundamentally determined by tumor phenotype, as defined by its underlying molecular architecture. The proliferative HCC-phenotype is characterized by upregulation of cell-cycle and mitotic genes (e.g., MCM6/7/8, BUB1B, and CDC6), progenitor-like features (e.g., CK-19 expression or Hoshida S2 signature), and elevated AFP levels, presenting a high risk of early recurrence [178,179,180]. Conversely, indolent tumors retain hepatocyte-like metabolic pathways and are associated with better outcomes. In line with this, high expression of Aldehyde Dehydrogenase 1 Family Member A1 (ALDH1A1) has been linked to minimal recurrence risk [180].

Similar principles are increasingly emerging in other liver malignancies, such as CCA and CRLM, where distinct genomic subclasses, actionable alterations, and immune microenvironment signatures appear to define divergent metastatic potential and hypothetical long-term benefit after transplantation [109,181].

Collectively, these data support a paradigm shift toward biologically driven and multidimensional selection models, integrating molecular tumor features with clinical variables to refine LT candidacy and optimize long-term oncologic outcomes. In this context, morphological-based criteria function more as technical “guard rails” than as precise predictors of post-LT outcomes, highlighting the imperfect correlation between tumor burden and the dynamic nature of its evolution [182,183].

In this scenario, elevated AFP levels reflect poor tumor differentiation and vascular invasion, translating into an increased risk of post-transplant recurrence [184,185].

An AFP threshold of 1000 ng/mL has been consistently identified as a critical prognostic cutoff, with patients below this value achieving markedly superior 5-year recurrence-free survival compared with those with higher levels (≈80% vs. ≈50%) [186]. Building on this evidence, several selection frameworks have combined AFP with morphologic parameters to refine risk stratification, such as the Hangzhou criteria, Metroticket 2.0 and the AFP-French score. These models have demonstrated survival outcomes comparable to Milan criteria while safely expanding transplant eligibility beyond conventional morphometrics, underscoring the added value of biological surrogates over size- and number-based selection alone [187,188,189,190,191].

Importantly, AFP kinetics have emerged as a more accurate predictor of post-transplant outcomes than static cutoffs. Longitudinal analyses have shown that rapid AFP increases during waiting-list time or failure to achieve AFP decline after therapy are strongly associated with early recurrence, whereas stable or decreasing AFP trajectories identify tumors with favorable biology [192,193,194]. Dynamic AFP-based models have therefore shifted selection paradigms from single-time-point thresholds toward continuous biological risk assessment.

Additional serum biomarkers reflective of tumor biology have been explored. Des-gamma-carboxyprothrombin (DCP), widely used in Asian transplant programs, has shown prognostic relevance when combined with AFP, enabling acceptable outcomes even in patients beyond Milan criteria [195,196,197]. More recently, dual-biomarker strategies incorporating AFP-L3 and DCP have demonstrated improved predictive accuracy for recurrence compared with AFP alone, although variability in measurement techniques and confounding clinical conditions currently limit their universal adoption [195]. Emerging evidence suggests that biomarkers related to angiogenesis, proliferation, and immune–inflammatory signaling, such as VEGF, MIB-1 proliferation index, E-cadherin level, and nuclear beta-catenin level, may further refine recurrence risk by reflecting the permissiveness of the host microenvironment [198,199].

However, heterogeneity in assay techniques, confounding clinical conditions, and limited external validation currently constrain their widespread clinical implementation.

Parallel to biomarker-based refinement, response to bridging and downstaging therapies has emerged as a powerful in vivo surrogate of tumor biology. Successful downstaging to within accepted criteria, followed by a mandatory observation period (“ablate-and-wait” strategy), identifies patients with low-risk of post-LT tumor recurrence [198,200,201].

Radiologic response assessed by modified RECIST criteria, complete pathological response on explant, and post-treatment AFP decline have all been independently associated with reduced recurrence risk [202,203,204].

Moreover, functional imaging and radiomics approaches, particularly FDG-PET and advanced CT- and MRI-derived features, have demonstrated the ability to capture metabolic activity, invasiveness, and microenvironmental heterogeneity associated with aggressive tumor behavior [205,206]. While their routine use in allocation policies remains limited by standardization and availability, these tools represent a promising bridge between morphologic assessment and biological risk stratification.

The next frontier lies in the application of machine learning–driven models, capable of integrating dozens of multidimensional variables, including AFP dynamics, radiomic features, inflammatory markers, and liver function indices, to outperform conventional scores in identifying patients at high risk of early recurrence [207] (voci). Collectively, this evolution reflects a conceptual transition from static, morphology-driven eligibility toward adaptive, biology-informed decision-making, aligning LT with the principles of precision transplant oncology.

#### 6.1.2. Integrating Host Immunity, Graft Injury, and Tumor Biology as Synergic Determinants of Post-Transplant Tumor Recurrence

Beyond tumor-intrinsic features, oncologic outcomes after LT are critically shaped by the dynamic interplay between residual tumor cells, the host immune system, and the transplanted allograft.

This complex host–tumor–graft crosstalk creates a unique biological environment where immune tolerance, inflammation, and tissue injury may collectively influence post-transplant recurrence.

LT induces a profound remodeling of the immune landscape, characterized by the establishment of allograft tolerance and long-term immunosuppression, which may inadvertently impair antitumor immune surveillance. Despite seemingly complete surgical removal of the primary lesion, residual circulating tumor cells (CTCs) or occult micro-metastases can exploit this permissive immune environment, engaging in bidirectional interactions with the allograft and immune cells that favor immune evasion and tumor persistence [208,209].

Recent studies demonstrated that post-transplant immune dysregulation involves alterations in both innate and adaptive immune compartments, including dysfunctional antigen presentation, expansion of regulatory T cells, and exhaustion of cytotoxic T lymphocytes, all of which contribute to reduced tumor immune control [208]. Immune exhaustion and senescence signatures, characterized by upregulation of inhibitory checkpoints and impaired effector function, have been associated with a two-fold increased risk of post-transplant malignancy, supporting the concept that the host immune state is a determinant of oncologic outcomes after LT [210].

Additionally, ischemia–reperfusion injury (IRI), an unavoidable consequence of prolonged cold and warm ischemia times during LT, represents a critical non–tumor-related driver of post-transplant tumor recurrence. Beyond its impact on graft function, IRI profoundly reshapes the hepatic microenvironment through the activation of hypoxia-responsive and inflammatory pathways, mediated by reactive oxygen species (ROS), damage-associated molecular patterns (DAMPs), and cytokine release, leading to endothelial activation, immune cell recruitment, and a pro-angiogenic microenvironment that may facilitate tumor cell survival and expansion [211].

Experimental and translational studies have shown that IRI also directly promotes tumor outgrowth by activating pro-tumorigenic signaling pathways, including IL-6/STAT3 and Th17-mediated immune responses, which enhance EMT, tumor cell invasiveness, and metastatic competence [212,213].

In line with this, Grąt et al. highlighted a critical association between the severity of IRI and early allograft dysfunction with a 1.5–2.5-fold higher risk of HCC-recurrence after LT, providing direct clinical evidence of the oncogenic consequences of graft injury [214]. In this context, emerging preservation and transplantation strategies, including hypothermic oxygenated perfusion (HOPE) and ischemia-free liver transplantation (IFLT), have shown promising results in attenuating IRI-driven inflammatory and angiogenic signaling [215]. By preserving endothelial integrity and limiting hypoxia-induced immune dysregulation, these approaches may contribute to oncologic risk reduction by reconditioning the post-transplant microenvironment, thereby aligning graft preservation techniques with the principles of precision transplant oncology [215].

The recipient’s pre-existing and post-transplant systemic inflammatory state represents another critical, yet often underestimated, determinant of tumor recurrence after LT [210,211].

Elevated inflammatory indices, including an increased neutrophil-to-lymphocyte ratio (NLR >3–4) and platelet-to-lymphocyte ratio (PLR), have been consistently validated as robust predictors of early post-transplant recurrence, reflecting a host milieu that favors metastatic seeding and tumor outgrowth [216,217,218,219,220]. The rationale is based on the perpetuation of systemic inflammation by the sustained release of DAMPs, such as mitochondrial DNA, which drives chronic immune activation. Over time, this persistent inflammatory signaling promotes immune exhaustion, characterized by the upregulation of inhibitory immune checkpoints (PD-1, TIM-3, LAG-3) on CD8^+^ T cells and natural killer cells, leading to impaired antitumor immune surveillance. This dysfunctional immune state is further reinforced by the expansion of myeloid-derived suppressor cells and regulatory T cells, which collectively establish an immunosuppressive niche within the graft [210].

Clinically, immune exhaustion and systemic inflammatory signatures have been associated with a 30–40% reduction in recurrence-free survival across multiple transplant cohorts, underscoring the role of systemic immune dysfunction as an active facilitator of post-transplant tumor recurrence rather than a passive epiphenomenon [210].

Together, these findings underscore that post-transplant oncologic outcomes are not solely dictated by tumor biology at the time of transplantation but are dynamically influenced by host immune competence, inflammatory burden, and graft-related injury. The convergence of immune tolerance, ischemia–reperfusion injury, and systemic inflammation creates a biologically permissive niche for tumor recurrence, reinforcing the need to integrate immunologic and graft-related parameters into contemporary risk stratification models within transplantation oncology.

### 6.2. Novel Biomarkers and Tools for Precision Risk Stratification in Transplant Oncology

Although the abovementioned attempts of introducing LT as a concrete and prognosis-changing opportunity for liver malignancies according to the molecular architecture, post-transplant tumor recurrence remains a significant challenge, affecting up to 20–50% of recipients, and is highly limited by the low sensitivity and specificity of current tools for cancer detection [221]. To address this problem, contemporary approaches aim at the development of new biomarker platforms to enable dynamic, longitudinal assessment of disease burden and treatment response, facilitating individualized risk stratification based on integrated molecular and genomic profiling beyond static tumor characteristics.

#### 6.2.1. Role of Circulating Tumor DNA (ctDNA)

In this setting, ctDNA has emerged as a non-invasive, promising liquid biomarker allowing for the real-time monitoring of tumor dynamics, detection of minimal residual disease, and monitoring rejection in patients undergoing LT [221].

In a recent study from Hong et al. examining a small cohort of patients undergoing LT for HCC, CCA, and CRLM, recipients with pre-transplant ctDNA positivity demonstrated higher absolute recurrence rates than those with negative ctDNA status. Moreover, sequential ctDNA testing showed that LT possesses the capacity to clear ctDNA, with 50% of patients achieving ctDNA clearance from positive to negative status [221].

Consistently, the ability of ctDNA in predicting early relapse and monitoring response to adjuvant treatment after definitive surgery has largely been demonstrated in HCC, with emerging data demonstrating that the persistent or emerging ctDNA positivity after LT is strongly associated with higher recurrence rates [222,223,224].

In a comprehensive analysis of 125 HCC patients with curative-intent treatments (including LT), ctDNA negativity in the molecular residual disease window (2–12 weeks post-transplantation) was achieved in 97.2% of patients who remained negative during subsequent surveillance, whereas all patients with detectable ctDNA at this critical timepoint experienced clinical recurrence with a hazard ratio of 7.2 [222,223,224].

This remarkable performance metric substantially exceeds traditional biomarkers such as AFP, with ctDNA demonstrating a median lead time of 7.9 months versus 2.2 months for AFP in predicting recurrence [222,223,224]. Interestingly, the mechanistic utility of ctDNA extends beyond simple presence-absence detection. Serial quantitative monitoring enables dynamic assessment of tumor evolution and treatment response, providing real-time windows into the changing genomic landscape during and after transplantation [225]. A landmark study integrating 721 plasma samples from HCC patients across multiple cancer stages documented that longitudinal ctDNA positivity demonstrated superior prognostic value compared to singular timepoint assessment, with sensitivities reaching 100% and a specificity of 97.8% for recurrence prediction when serial monitoring was performed [225]. Notably, the median lead time from initial ctDNA detection to radiologically confirmed recurrence was 3.5 months, enabling potential interventions during a clinically actionable window. Furthermore, evidence from secondary liver malignancies, including CRLM, demonstrates that 75% of patients with post-operative ctDNA positivity experienced clinical relapse within 6 months, with most achieving oligometastatic disease amenable to repeat hepatic resection [225,226]. In CCA, although data remain limited, small series suggest that tumor-informed ctDNA assays may anticipate radiologic relapse before imaging detection [227].

Collectively, these data support ctDNA as a transformative biomarker in transplant oncology, capable of capturing tumor biology with a sensitivity and temporal resolution that surpass conventional radiologic and serologic surveillance.

By identifying minimal residual disease during the critical post-transplant window, stratifying recurrence risk before clinical manifestation, and dynamically tracking tumor evolution under immunosuppression, ctDNA offers a unique opportunity to shift from reactive to anticipatory management. Prospective, transplant-specific validation studies are now warranted to define standardized thresholds, optimal timing, and integration algorithms.

#### 6.2.2. Spatial Transcriptomics and Multi-Omics Approaches

Spatial transcriptomics has revolutionized our understanding of cell interactions and microenvironmental organization in liver malignancies [228,229]. By preserving tissue architecture while quantifying gene expression, the integration of single-cell RNA sequencing with spatial transcriptomics has identified highly localized communication patterns between distinct cell populations, revealing how spatial organization governs tumor fitness, immune evasion, and therapeutic responsiveness [230,231,232].

To date, the most comprehensive spatial evidence derives from HCC studies, where immune infiltration is highly compartmentalized rather than uniformly distributed [233]. Chemokine-defined niches organize functional immune territories: CCL15-enriched tumor cores preferentially recruit CD163^+^ M2-like macrophages, fostering immunosuppressive environments associated with adverse prognosis, whereas peripheral regions enriched in CCL19 and CCL21 promote structured lymphoid aggregates containing CD3^+^ T cells and CD20^+^ B cells, correlating with improved outcomes [233]. These observations underscore that immune competence is spatially orchestrated and that architectural organization, not merely immune density, contributes to the biological behavior.

Consistently, in HCC patients treated with atezolizumab plus bevacizumab, Lim J et al. demonstrated that responders presented an immune-activated tumor microenvironment characterized by coordinated cytotoxic T-cell clusters, inflammatory macrophage populations, enhanced dendritic cell interactions, and preserved antigen-presenting function, whereas non-responders displayed spatially restricted immunosuppressive niches with impaired immune cross-talk [234]. In line with this, in HCC with microvascular invasion, invasive fronts contain proliferative tumor clones spatially associated with TREM2^+^ macrophages, LAMP3^+^ dendritic cells, activated myofibroblasts, and cycling T cells [235].

Although spatial mapping is less mature in CCA and CRLM, emerging data indicate that similar principles also apply in these settings. In iCCA, dense desmoplastic stroma and CAF-rich regions establish physical and biochemical barriers to immune infiltration, contributing to immune exclusion phenotypes [109]. In CRLM, spatial organization of immune infiltrates at the invasive margin, rather than absolute immune cell counts, has been associated with recurrence risk and treatment response [236]. Collectively, these findings suggest that spatially encoded tumor–immune–stroma interactions represent a shared determinant of aggressiveness across primary and secondary liver malignancies.

From a translational perspective, spatial profiling may offer a complementary layer of biological risk stratification in transplant oncology. Tumors characterized by immune-excluded architectures, aggressive invasive niches, or stromal-dominated immunosuppressive programs may harbour a higher intrinsic risk of post-transplant recurrence despite meeting conventional morphological criteria [237]. Conversely, tumors exhibiting organized immune-permissive territories and preserved cytotoxic niches may represent biologically favorable subsets [238]. Integration of spatial signatures with immune repertoire profiling could therefore refine candidate selection and inform personalized surveillance or immunosuppressive modulation strategies following LT.

Future paradigms will likely rely on integrated multi-omics profiling, combining tumor genomics, immune repertoire analysis, ct-DNA, and donor-derived cell-free DNA to dynamically guide immunomodulatory strategies [210,239]. Such approaches may enable real-time adjustment of immunosuppression in response to early signals of rejection or tumor recurrence, establishing a precision medicine framework for transplant oncology.

Table 2 summarizes the emerging tools for tailored risk stratification in transplant oncology (Table 2).

## 7. Conclusions

In conclusion, LT for hepatic malignancies is undergoing a profound conceptual transformation. The traditional paradigm based on rigid morphological criteria is progressively being replaced by a more nuanced approach that integrates tumour biology, treatment response, and patient-specific factors. This shift has already expanded transplant eligibility in selected patients with hepatocellular carcinoma and is increasingly being explored in cholangiocarcinoma and colorectal liver metastases, where historically transplantation was considered contraindicated.

From a clinical perspective, these developments highlight the importance of multidisciplinary evaluation, biologically driven candidate selection, and the integration of systemic and locoregional therapies as part of dynamic treatment strategies. In particular, emerging approaches such as immunotherapy-based downstaging, molecular profiling, and advanced imaging are contributing to refining patient selection, although their routine implementation remains limited by the lack of prospective validation.

Looking forward, future research should focus on the development of standardized selection criteria incorporating biological and molecular markers, optimization of timing and sequencing of neoadjuvant therapies, and definition of tailored immunosuppressive strategies in the context of prior immunotherapy exposure. In parallel, the expansion of the donor pool through machine perfusion and extended-criteria donors may play a crucial role in supporting broader access to transplantation. Prospective multicentre studies and collaborative registries will be essential to validate these evolving strategies and to ensure that the expansion of transplant oncology remains both clinically effective and ethically sustainable.

## Figures and Tables

**Table 1:** Evolution of Liver Transplantation Strategies in Cholangiocarcinoma (CCA).

Domain	Perihilar CCA (pCCA)	Intrahepatic CCA (iCCA)	Biological Downstaging and Targeted Therapy	Ref.
**Clinical context**	Frequently unresectable due to vascular involvement and biliary extension	Often diagnosed late; high recurrence after LT	Advanced disease with actionable molecular alterations	[108,109,110]
**Role of LT**	Established in highly selected patients	Investigational; limited to clinical trials	Emerging strategy to expand LT candidacy	[110,111,118,119,120,122,123,124]
**Key protocols/studies**	Mayo Clinic protocol; validated multicenter experience	Sapisochin et al.; TESLA, iCOLA, LIRICA trials	ClarIDHy trial; OPT-IC trial; pemigatinib case reports	[118,119,120,122,123,135,137,141]
**Neoadjuvant strategy**	Chemoradiation (EBRT + fluoropyrimidines) + brachytherapy + maintenance chemotherapy	Systemic chemotherapy ± immunotherapy + TARE (SYS-TARE)	Targeted therapy (FGFR, IDH1 inhibitors) ± chemotherapy	[110,124,132,133,134,135,136,137]
**Selection criteria**	UNOS: tumor ≤ 3 cm, no metastases, no lymph nodes, stable disease	Very early tumors (<2 cm), stable disease after systemic therapy	Molecular selection (FGFR2, IDH1/2, BRAF) and response	[109,122,126,135,142,143,144,145]
**Biological selection role**	Dropout during waiting list reflects aggressive disease	Tumor size and response predict outcomes	Response to targeted therapy as biological test	[122,126,135]
**Outcomes**	5-year OS ~60–70%	Promising in early-stage cases; limited evidence	Early tumor regression; potential expansion of eligibility	[110,122,124,126,127,128,135]
**Allocation strategies**	MELD exception (MMaT-3); improved access	Under evaluation in trials	Not standardized	[130]
**LDLT role**	Reduces dropout; promising results	Explored in innovative protocols	Enables expansion despite organ shortage	[131,138,141]
**Limitations**	Strict selection; ~5% eligible; complexity	High recurrence risk; small cohorts	Limited data; lack of trials	[122,126,135]
**Future directions**	Refinement of selection and allocation	Biomarker-driven selection; genomics integration	Neoadjuvant targeted therapy; MRD monitoring	[130]

CCA: cholangiocarcinoma; pCCA: perihilar cholangiocarcinoma; iCCA: intrahepatic cholangiocarcinoma; LT: liver transplantation; LDLT: living donor liver transplantation; UNOS: United Network for Organ Sharing; MELD: Model for End-Stage Liver Disease; MMaT-3: Median MELD at Transplant minus 3; EBRT: external beam radiotherapy; TARE: transarterial radioembolization; SYS-TARE: systemic therapy combined with transarterial radioembolization; OS: overall survival; FGFR: fibroblast growth factor receptor; IDH: isocitrate dehydrogenase; BRAF: B-Raf proto-oncogene; MRD: minimal residual disease.

**Table 2:** Emerging biomarkers and tools for precise risk stratification in transplant oncology.

Domain	Circulating Tumor DNA (ctDNA)	Spatial Transcriptomics	Integrated Multi-Omics Approaches	Ref.
Concept	Liquid biopsy for real-time tumor monitoring	Spatial mapping of gene expression within tissue architecture	Integration of genomics, immune profiling, and circulating biomarkers	[210,221,230,231,232,239]
Clinical utility	Detection of minimal residual disease (MRD); recurrence prediction; monitoring response	Characterization of tumor microenvironment and immune niches	Dynamic risk stratification and personalized management	[109,210,221,222,223,224,228,234,236,237,238,239]
Key evidence	Higher recurrence with pre-LT ctDNA positivity; HR ~7.2 for recurrence; lead time up to 7.9 months vs. AFP	Immune niches (CCL15, CCL19/21) correlate with prognosis; spatial immune patterns predict response	Emerging studies integrating ctDNA, genomics, and immune data	[210,222,223,224,228,239]
Temporal advantage	Early detection before radiologic recurrence (3.5–7.9 months lead time)	Captures spatial heterogeneity rather than temporal dynamics	Real-time longitudinal monitoring	[210,222,223,224,225,228,229,230,231,232]
Biological insight	Tracks tumor evolution and clonal dynamics	Reveals tumor–immune–stroma interactions	Combines systemic and local biological information	[109,221,234,235,236]
Applications in LT	Pre- and post-transplant recurrence risk assessment; MRD detection	Identification of high-risk tumor architectures	Guiding immunosuppression and surveillance strategies	[210,221,225,228,235,236]
Limitations	Need for standardization; cost; limited transplant-specific validation	Limited data in CCA and CRLM; technical complexity	Integration complexity; lack of standardized algorithms	[109,210,221,236,239]
Future directions	Prospective validation; threshold definition; integration in clinical workflows	Expansion to CCA and CRLM; integration with immunotherapy response	Precision transplant oncology; adaptive immunosuppression	[109,210,221,236,239]

Abbreviations: LT, liver transplantation; ctDNA, circulating tumor DNA; MRD, minimal residual disease; AFP, alpha-fetoprotein; CCA, cholangiocarcinoma; CRLM, colorectal liver metastases.

## Data Availability

No new data were created or analyzed in this study.

## Abbreviations

The following abbreviations are used in this manuscript:

AFP	Alpha-Fetoprotein
AI	Artificial Intelligence
ATG	Anti-Thymocyte Globulin
BRAF	B-Raf Proto-Oncogene
CAFs	Cancer-Associated Fibroblasts
CCA	Cholangiocarcinoma
CEA	Carcinoembryonic Antigen
CNIs	Calcineurin Inhibitors
CRC	Colorectal Cancer
CRLM	Colorectal Liver Metastases
CTCs	Circulating Tumor Cells
ctDNA	Circulating Tumor DNA
CTLA-4	Cytotoxic T-Lymphocyte-Associated Antigen-4
DFS	Disease-Free Survival
EBRT	External Beam Radiotherapy
EGFR	Epidermal Growth Factor Receptor
FGFR	Fibroblast Growth Factor Receptor
FLR	Future Liver Remnant
GemCis	Gemcitabine–Cisplatin
HCC	Hepatocellular Carcinoma
iCCA	Intrahepatic Cholangiocarcinoma
ICIs	Immune Checkpoint Inhibitors
IDH	Isocitrate Dehydrogenase
KRAS	Kirsten Rat Sarcoma Viral Oncogene Homolog
LAG-3	Lymphocyte Activation Gene-3
LT	Liver Transplantation
MASLD	Metabolic Dysfunction-Associated Steatotic Liver Disease
MDSCs	Myeloid-Derived Suppressor Cells
MELD	Model for End-Stage Liver Disease
MMaT-3	Median MELD at Transplant minus 3
MRD	Minimal Residual Disease
MSI	Microsatellite Instability
mTOR	Mechanistic Target of Rapamycin
MWA	Microwave Ablation
NGS	Next-Generation Sequencing
OS	Overall Survival
pCCA	Perihilar Cholangiocarcinoma
PD-1	Programmed Cell Death Protein-1
PD-L1	Programmed Death-Ligand 1
PIVKA-II	Protein Induced by Vitamin K Absence-II
PLC	Primary Liver Cancer
RFA	Radiofrequency Ablation
SBRT	Stereotactic Body Radiotherapy
SMAD4	SMAD Family Member 4
SYS-TARE	Systemic Therapy plus TARE
TACE	Transarterial Chemoembolization
TAMs	Tumor-Associated Macrophages
TARE	Transarterial Radioembolization
TIM-3	T-cell Immunoglobulin and Mucin-domain Containing-3
TME	Tumor Microenvironment
TMB	Tumor Mutational Burden
TP53	Tumor Protein 53
Tregs	Regulatory T Cells
UNOS	United Network for Organ Sharing
VEGF	Vascular Endothelial Growth Factor
